# Bronchial extracellular matrix from COPD patients induces altered gene expression in repopulated primary human bronchial epithelial cells

**DOI:** 10.1038/s41598-018-21727-w

**Published:** 2018-02-22

**Authors:** Ulf Hedström, Oskar Hallgren, Lisa Öberg, Amy DeMicco, Outi Vaarala, Gunilla Westergren-Thorsson, Xiaohong Zhou

**Affiliations:** 1 Bioscience Regeneration Department, Respiratory, Inflammation and Autoimmunity, IMED Biotech Unit, AstraZeneca, Gothenburg, Sweden; 2 Division of Lung Biology, Department of Experimental Medical Science, Lund University, Lund, Sweden; 3 Division of Respiratory Medicine and Allergology, Department of Clinical Sciences, Lund University, Lund, Sweden; 4 Bioscience Immunity Department, Respiratory, Inflammation and Autoimmunity, IMED Biotech Unit, AstraZeneca, Gothenburg, Sweden

## Abstract

Chronic obstructive pulmonary disease (COPD) is a serious global health problem characterized by chronic airway inflammation, progressive airflow limitation and destruction of lung parenchyma. Remodeling of the bronchial airways in COPD includes changes in both the bronchial epithelium and the subepithelial extracellular matrix (ECM). To explore the impact of an aberrant ECM on epithelial cell phenotype in COPD we developed a new *ex vivo* model, in which normal human bronchial epithelial (NHBE) cells repopulate and differentiate on decellularized human bronchial scaffolds derived from COPD patients and healthy individuals. By using transcriptomics, we show that bronchial ECM from COPD patients induces differential gene expression in primary NHBE cells when compared to normal bronchial ECM. The gene expression profile indicated altered activity of upstream mediators associated with COPD pathophysiology, including hepatocyte growth factor, transforming growth factor beta 1 and platelet-derived growth factor B, which suggests that COPD-related changes in the bronchial ECM contribute to the defective regenerative ability in the airways of COPD patients.

## Introduction

Chronic obstructive pulmonary disease (COPD) is characterized by chronic airway inflammation, loss of small airways and emphysema^1^, leading to a progressive and largely irreversible airflow limitation^2^. It is a considerable global health problem and is projected to be the fourth leading cause of death worldwide by the year 2030^3^. Tobacco smoke is the primary risk factor behind COPD and smoking cessation is considered one of the most important preventive measures, but there is a large unmet medical need among patients that have developed COPD. The airway epithelium constitutes the first line of defense in the lungs and plays a crucial role in protection against microbes, noxious gases and other inhaled environmental insults. This defense barrier is functionally impaired in COPD patients and pathological features such as squamous metaplasia^4^, goblet cell hyperplasia^5^ and decreased epithelial integrity^6^ leads to reduced mucociliary clearance, excess mucus production and an increased susceptibility to respiratory infections.

The extracellular matrix (ECM) is made up of a complex macromolecular network of proteins and proteoglycans providing both rigidity and flexibility to the tissue structure of all organs. However, beyond acting as a structural support for the cells, the ECM also has important regulatory functions, influencing cell proliferation, differentiation and migration^7^. One way by which the ECM can modulate cell function is by acting as a reservoir for growth factors and inflammatory mediators^8,9^. Several studies have shown that the ECM in the central airways is remodeled in COPD^10–12^. Gaining a better understanding of how a diseased ECM modulates epithelial cell phenotype in COPD could provide more insight into mechanisms of pathological remodeling of the airway epithelium, which may expand possibilities for pharmacological intervention.

We hypothesized that pathological changes in the bronchial ECM drive remodeling of the airway epithelium in COPD patients and the aim of this study was to better understand the role of the bronchial ECM during epithelial cell differentiation. A new *ex vivo* model was therefore developed in which normal human bronchial epithelial (NHBE) cells repopulate and differentiate on decellularized bronchial scaffolds from patients with severe COPD and healthy donors. The model was used to study the impact of ECM on bronchial epithelial cell phenotype, with respect to differentiation, proliferation, apoptosis and global gene expression.

Using this model we show for the first time that bronchial ECM derived from COPD patients has an impact on gene expression in airway epithelial cells early during the differentiation process. The differential gene expression pattern indicates altered activity of upstream mediators involved in regeneration and remodeling, such as hepatocyte growth factor (HGF), transforming growth factor beta 1 (TGF-β1) and platelet-derived growth factor B (PDGF-BB). Our data also demonstrate that this new *ex vivo* model offers a promising platform for studying the impact of bronchial ECM on airway epithelial cells and the potential factors in the ECM contributing to the defective regenerative ability in the airways of COPD patients.

## Materials and Methods

### Tissue acquisition

Lungs from 3 patients with severe COPD (GOLD stage IV) and 3 healthy donors (including one ex-smoker) were acquired from the Department of Cardiothoracic Surgery at Sahlgrenska University Hospital in Gothenburg. The study was approved by the Swedish Research Ethical Committee in Gothenburg (FEK 675-12/2012) and in Lund (FEK 91/2006) and performed in accordance with the Helsinki Declaration. No organs or tissues in this study were procured from prisoners and informed consent was obtained from all subjects or their closest relatives.

### Decellularization

Bronchial airways (2^nd^–4^th^ segment) were dissected from the lungs, frozen in liquid nitrogen and stored at −80 °C. A cryostat (Microm HM 560) was used to cut the airways into 500 µm thick cryosections, which were immediately placed in phosphate-buffered saline (PBS) at room temperature (RT). Any remaining parenchyma was removed and decellularization (DC) was performed by treating the sections with the following solutions: 4% (w/v) sodium deoxycholate (Sigma-Aldrich 30970) for 2.5 hours (the solution was changed every 30 minutes), Hank’s Balanced Salt Solution for 3 × 5 minutes, 1000 Kunitz units/ml of deoxyribonuclease I (DNase I) (Sigma-Aldrich D4527) with 0.5 mM CaCl_2_ for 60 minutes and PBS for 3 × 5 minutes. All DC steps were done at RT on an orbital shaker set to 170 rpm, except the DNase I incubation, which was done at 37 °C without agitation. The decellularized scaffolds were stored in PBS at 4 °C for up to 2 days before being used for repopulation. All PBS used during sectioning and DC had been supplemented with 50 U/ml penicillin, 50 µg/ml streptomycin, 50 µg/ml gentamicin and 2 µg/ml amphotericin B.

### Quantification of DNA, sulfated glycosaminoglycans and elastin

Non-decellularized and decellularized bronchial airway tissue was dried at 50 °C for 2.5 hours, followed by weighing, before extraction of DNA, glycosaminoglycans (GAGs) or elastin. DNA was extracted using the DNeasy Blood & Tissue Kit (Qiagen 69504) and quantified using the Quant-iT PicoGreen dsDNA Assay Kit (Thermo Fisher P11496) (n = 6). Sulfated GAGs and soluble α-elastin were extracted and quantified using the Blyscan Sulfated GAG (Biocolor B1000) and Fastin (Biocolor F2000) Assay Kits, respectively, all according to the manufacturer’s instructions (n = 3). DNA, sulfated GAG and α-elastin concentrations in the extracts were normalized against dry tissue weight.

### Cell culture, repopulation and differentiation

Primary NHBE cells from a single donor were purchased from Lonza and cultured in Bronchial Epithelial Cell Growth Medium (BEGM) (Lonza CC-3170) before being frozen in passage 2. The cells were thawed and cultured in BEGM for 6 days with a medium change every 2–3 days. On the day of repopulation the cells had a confluence of ~90% and were detached from the flasks using StemPro Accutase Cell Dissociation Reagent (Thermo Fisher A1110501), centrifuged at 300 × g for 5 minutes, resuspended in BEGM and counted using a Nucleocounter NC-200 (Chemometec).

The decellularized scaffolds were carefully placed on top of sterile polycarbonate Whatman filters (Sigma-Aldrich WHA110614), which were transferred to 6-well plates filled with BEGM, allowing them to float on the medium surface. Primary NHBE cells were carefully dispensed on top of the scaffolds, which were then incubated with the cells at 37 °C. On the subsequent day, 75% of the medium in each well was replaced with fresh BEGM.

Differentiation was induced four days after the addition of cells to the scaffolds by exchanging the BEGM for a differentiation medium and carefully removing excess medium immediately surrounding the scaffolds. The day differentiation was initiated was defined as day 0. The differentiation medium was composed of 50% (v/v) BEGM Stock Solution, 50% (v/v) Dulbecco’s Modified Eagle’s Medium (DMEM) (Thermo Fisher 41965) Stock Solution and 0.05 µM retinoic acid (Sigma-Aldrich R2625). The DMEM Stock Solution had previously been supplemented with 1 mM sodium pyruvate, 2 mM L-glutamine and Minimal Essential Medium Non-Essential Amino Acids Solution (Thermo Fisher 11360, 25030 & 11140, respectively) at working concentration. All the included BEGM supplements had been added to the BEGM Stock Solution at 2 times the working concentration except for retinoic acid, which had been omitted. The scaffolds were cultured with differentiation medium for up to 35 days with a medium change every 2–3 days. New differentiation medium with freshly added retinoic acid was prepared from the BEGM and DMEM Stock Solutions before each medium change. As markers of differentiation, we used FoxJ1 (ciliated cells), mucin 5AC (MUC5AC) (goblet cells), p63 (basal cells) and ZO-1 (tight junctions).

**Figure 1:**
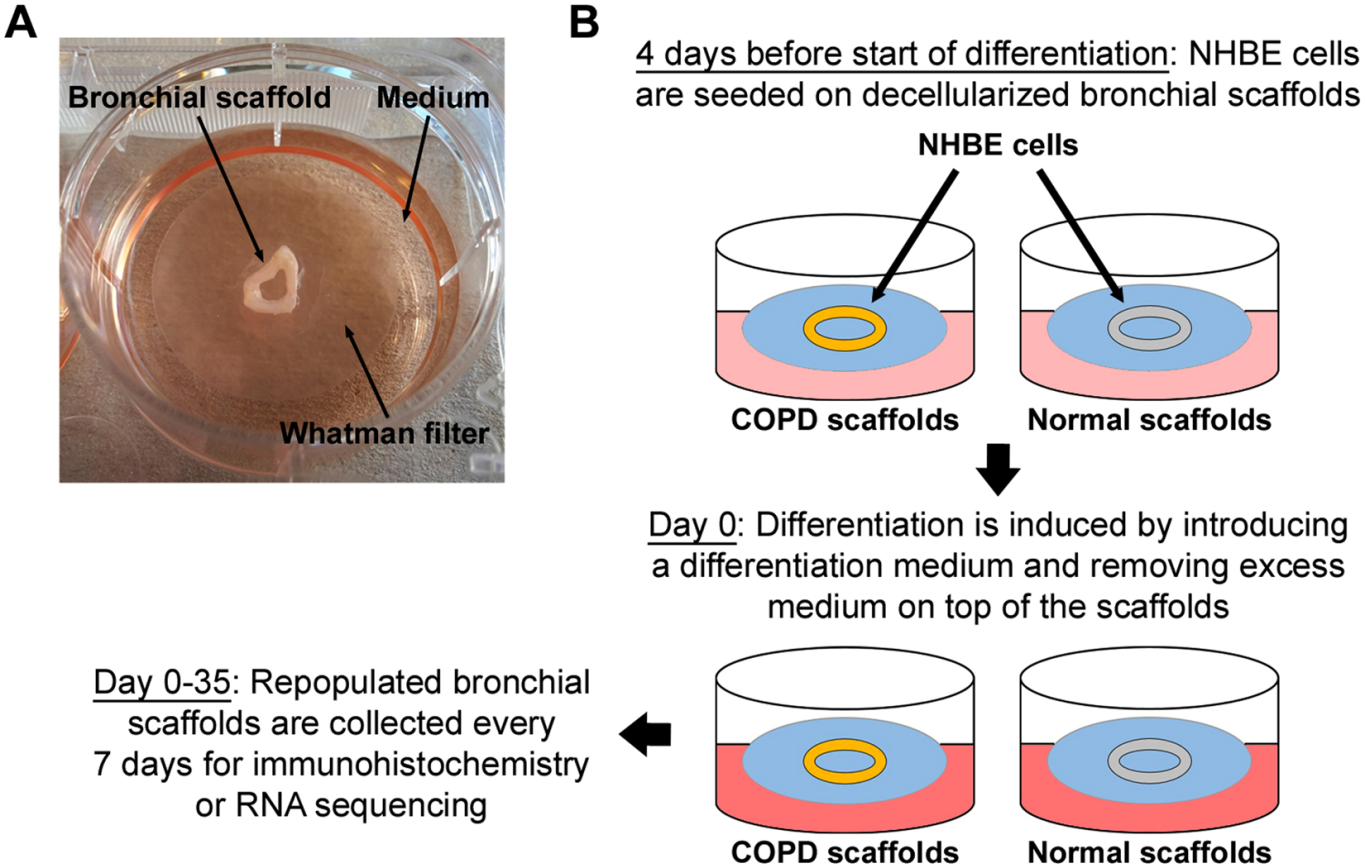
Experimental setup for *ex vivo* model used for repopulating bronchial scaffolds with primary normal human bronchial epithelial cells. (**A**) After decellularization the bronchial scaffolds are placed on sterile Whatman filters floating on medium in 6-well plates. This setup allows the cells to differentiate close to an air-liquid interface. (**B**) Primary normal human bronchial epithelial (NHBE) cells at passage 3 are seeded on decellularized bronchial scaffolds derived from COPD patients and healthy donors. Four days later the medium used during seeding is replaced with a differentiation medium and excess medium remaining on the scaffolds is removed to maximize exposure to air. Repopulated scaffolds are collected for immunohistochemistry or RNA sequencing on day 0, 7, 14, 21, 28 and 35.

Repopulated scaffolds were collected at different time points. For histology, TUNEL staining and immunohistochemistry (IHC), scaffolds (n = 3) were fixed in 4% formaldehyde for 20–24 hours at RT. Scaffolds designated for RNA isolation (n = 3) were snap frozen in liquid nitrogen and stored at −80 °C. Repopulated scaffolds for histology/IHC were seeded with 0.3 million NHBE cells/scaffold and the ones for RNA isolation with 0.15 million NHBE cells/scaffold. The experimental setup is visualized in Fig. 1.

### Immunohistochemistry, histology and TUNEL staining

Fixed bronchial scaffolds were embedded in histogel (Thermo Fisher HG-4000) and dehydrated in ethanol and xylene, followed by embedding in paraffin. 4 µm thick sections were made using a microtome (Leica RM2165). Before staining, the sections were deparaffinized in xylene and rehydrated in ethanol followed by deionized water.

IHC was performed as previously described^13^, but with 5% normal goat serum added to the blocking buffer and incubation with primary antibodies overnight at 4 °C. All primary antibodies and epitope retrieval methods used for IHC are shown in Table 1. Mouse IgG isotype control or rabbit Ig fraction were used as negative controls. Heat induced epitope retrieval (HIER) was done in citrate (pH 6) or Tris/EDTA (pH 9) buffer. Epitope retrieval with proteinase K was done for 15 minutes at 37 °C and with heparinase III overnight at RT. For double staining against p63 and FoxJ1, sections were blocked with mouse IgG blocking reagent (M.O.M. kit, Vector Laboratories BMK-2202) after the FoxJ1 staining to prevent cross reactivity during the subsequent p63 staining. The p63 and FoxJ1 positive cells were detected with streptavidin-conjugated Alexa 488 and 568, respectively. Biotinylated goat anti-mouse or anti-rabbit IgG (Vector Laboratories) was used and all antibodies were diluted in blocking buffer. Masson’s trichrome staining was performed using a Masson’s Trichrome Stain Kit (Sigma-Aldrich HT15) according to the manufacturer’s instructions. Sections were stained with hematoxylin and eosin and alcian blue-periodic acid Schiff (AB-PAS) using standard protocols. TUNEL (TdT-mediated dUTP Nick-End Labeling) staining was performed using the DeadEnd Colorimetric TUNEL kit (Promega G7130), according to the manufacturer’s instructions. Native bronchial tissue sections, treated with DNase I or with the TUNEL labeling enzyme TdT (Terminal Deoxynucleotidyl Transferase) omitted, were used as positive and negative controls, respectively.

**Table 1:** Primary antibodies and epitope retrieval methods used for immunohistochemistry.

Antibody	Species	Supplier	Catalog number	Concentration	Epitope retrieval
Collagen IV	Rabbit	Abcam	ab6586	0.5 µg/ml	HIER at pH 6
Laminin	Rabbit	Agilent Technologies	Z0097	10 µg/ml	Proteinase K
Perlecan	Mouse	Thermo Fisher	13–4400	3 µg/ml	Heparinase III
FoxJ1	Mouse	eBioscience	14–9965	2.5 µg/ml	HIER at pH 6
MUC5AC	Mouse	Thermo Fisher	MS-145-P1	1 µg/ml	HIER at pH 6
p63	Mouse	Agilent Technologies	M7317	1 µg/ml	HIER at pH 9
Ki-67	Mouse	Agilent Technologies	M7240	0.6 µg/ml	HIER at pH 6
ZO-1	Mouse	BD Biosciences	610966	5 µg/ml	HIER at pH 6

HIER = Heat induced epitope retrieval.

After IHC, histology and TUNEL staining the sections were dehydrated in ethanol and xylene, mounted with Pertex mounting medium (Histolab 00811) and photographed using an Olympus BX50F microscope equipped with an Olympus DP80 camera. Sections double stained for p63/FoxJ1 were not dehydrated, but immediately mounted in Vectashield medium with DAPI (Vector Laboratories). Unless stated otherwise, all IHC and histology staining steps were done at RT.

### Image analysis

All IHC and TUNEL stained sections from repopulated scaffolds were scanned at 20 times magnification using a Zeiss Axio Scan.Z2 scanner. The virtual slides were imported into the Visiopharm Integrator System 6.0 software. Regions of interest in each tissue section were defined manually. All cells seen on the luminal side of each repopulated scaffold section were included in the image analysis. Counterstaining with hematoxylin allowed for counting of the total number of cells. The fraction of positive cells for each marker was calculated using Analysis Protocol Packages, which are protocols in the Visiopharm software that classify cells as positive or negative. At least 400 cells were counted per patient/donor and time point for each marker.

### RNA extraction, cDNA library synthesis and RNA sequencing

Repopulated scaffolds were disrupted and homogenized for 3 × 1 minutes using a TissueLyser II bead mill (Qiagen) set to 30 Hz, followed by total RNA extraction using the RNeasy Mini Kit (Qiagen 74104), including on-column DNase I digestion. RNA integrity was evaluated with a 2100 Bioanalyzer system (Agilent Technologies) and the RNA was quantified using the Quant-iT RiboGreen RNA Assay Kit (Thermo Fisher R11490).

RNA was diluted to 10 ng/µl and used as input to create cDNA libraries using a TruSeq Stranded mRNA Library Preparation kit (Illumina RS-122-2103) with dual indexing following the manufacturer’s instructions. Libraries were validated on the Fragment Analyzer Automated CE platform (Advanced Analytical) using the standard sensitivity NGS fragment analysis kit and the concentration was determined using Quant-iT dsDNA High Sensitivity assay kit on the Qubit fluorometer (Thermo Fisher). Sample libraries were pooled in equimolar concentrations and diluted and denatured according to Illumina guidelines. RNA Sequencing (RNA-Seq) was performed using a High Output 1 × 76 bp kit on an Illumina NextSeq 500 platform.

### Bioinformatic analysis

RNA-Seq fastq files were processed using bcbio-nextgen (version 1.0.1) (https://github.com/chapmanb/bcbio-nextgen) where reads were mapped to the human genome build hg38 (GRCh38.79) using hisat2 (version 2.0.5)^14^ yielding between 5.3–20.6 M mapped reads (10.8 M on average) with a 97% mapping frequency or higher per sample. Gene level quantifications and counts were generated with featurecounts (version 1.4.4)^15^ within bcbio-nextgen. ArrayStudio version 9 (OmicSoft, Cary, NC) was used for further data analysis. Heatmaps were created based on hierarchical clustering of log2-transformed ratios (COPD/normal) of DESeq2-normalized counts^16^, using correlation as the similarity measure. Data for individual genes were plotted using log2-transformed DESeq2-normalized counts. The scientific literature based commercial software package Ingenuity Pathway Analysis (Qiagen Inc.) (https://www.qiagenbioinformatics.com/products/ingenuity-pathway-analysis) was used for upstream mediator analysis^17^ with an absolute activation z score >2 and a p value < 0.001 (Fisher’s exact test) as cut-offs for significance.

### Real-time quantitative RT-PCR

Synthesis of cDNA was done with the High Capacity RNA to cDNA kit (Applied Biosystems) according to the manufacturer’s instructions. Real-time quantitative Reverse Transcription Polymerase Chain Reaction (qRT-PCR) was carried out using Taqman Fast Advanced Master Mix and Taqman primers and probes specific to cDNAs of interest (Applied Biosystems), and data were acquired on the QuantStudio 7 Flex system. Relative expression was normalized against expression of TBP (TATA box binding protein) mRNA and calculated as 2^-(Ct_SAMPLE_ − Ct_TBP_).

### Statistics

Analyses of RNA-Seq data were performed with DESeq2^16^, using raw counts as input, and genes were considered significantly differentially expressed if they had a False Discovery Rate (FDR) value < 0.05 using the Benjamini-Hochberg method for multiple comparison correction. DNA, sulfated GAG and elastin data were analyzed with the Mann-Whitney test. Quantitative IHC/TUNEL data as well as real-time qRT-PCR data were analyzed with two-way ANOVA tests using Sidak correction. All analyses were done using GraphPad Prism 7.02 and differences were considered significant if p < 0.05.

### Data availability

The complete RNA-Seq data set is available in the Gene Expression Omnibus data repository (https://www.ncbi.nlm.nih.gov/geo) and is accessible through GEO Series accession number GSE107971. All other data sets in the study are included in this published article and its supplementary information files.

## Results

### Decellularization of human bronchial airways efficiently removes cells while preserving the extracellular matrix

**Figure 2:**
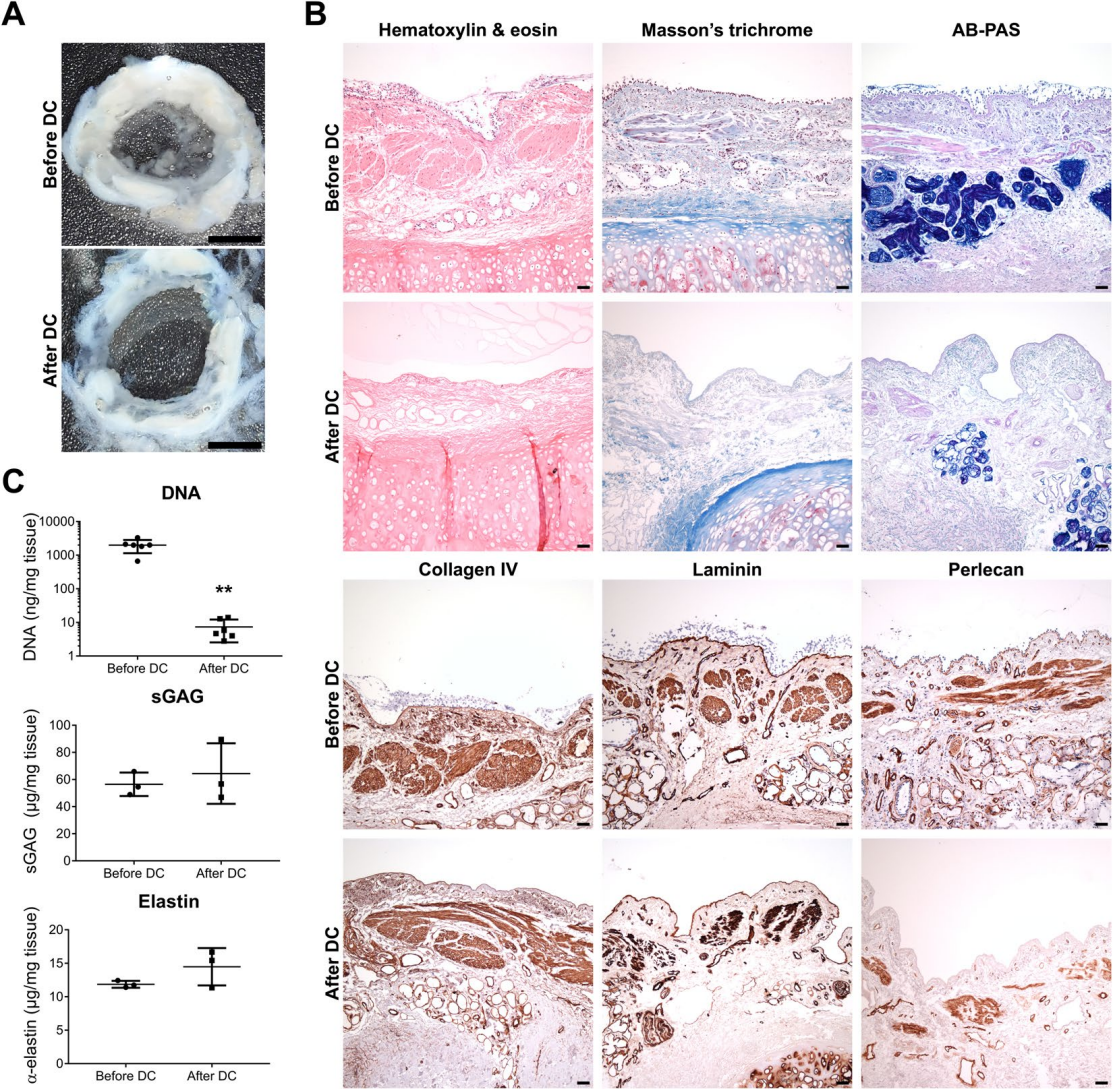
Decellularization of human bronchial airways efficiently removes cells while preserving the extracellular matrix. (**A)** The macroscopic structure of the 500 µm thick human bronchial tissue sections was preserved after decellularization (DC). **(B)** Hematoxylin & eosin, Masson’s trichrome (collagen in blue) and alcian-blue periodic acid Schiff (AB-PAS) (polysaccharides in magenta/purple) stainings as well as immunohistochemistry against the basement membrane proteins collagen IV, laminin and perlecan, before and after DC. Images are representative of n = 3. **(C)** Dye-binding methods confirmed that DC efficiently decreased DNA content (**p = 0.002) while preserving sulfated glycosaminoglycans (sGAG) and elastin in bronchial scaffolds (n = 6 for DNA, n = 3 for sGAG and elastin). The data were analyzed with the Mann-Whitney test and graphs indicate mean and standard deviation. Scale bars: 3 mm in A and 50 µm in B.

The macroscopic tissue structure of the bronchial cryosections was preserved after DC (Fig. 2A). Hematoxylin and eosin staining confirmed the absence of nuclei and revealed a preserved tissue structure in the bronchial scaffolds (Fig. 2B). The epithelial basement membrane (BM) as well as the ECM of the lamina propria and submucosa could be clearly seen also after DC. Occasional remaining nuclei were observed in the lacunae of the cartilage. Masson’s trichrome and AB-PAS staining demonstrated preservation of collagens and polysaccharides, respectively, in the ECM after DC (Fig. 2B). IHC showed that collagen IV and laminin remained in the scaffolds after DC and the staining for both was found in BMs. The staining pattern for perlecan IHC indicated that it was not fully preserved in all BMs after DC and the expression was weaker in the epithelial BM compared to the BMs representing the ECM remnants of blood vessels and the smooth muscle layer (Fig. 2B).

DNA measurements confirmed that DC efficiently decreased DNA content in the scaffolds (Fig. 2C), but no reduction was seen for sulfated GAGs or elastin. Non-significant increases were seen for the averages of both sulfated GAGs and elastin after DC, which may be due to a reduction in tissue weight caused by removal of cells. The DNA results met the standard for tissue DC as reported by Crapo *et al*.^18^.

### Primary human bronchial epithelial cells develop cilia and assume a pseudostratified morphology during differentiation on bronchial scaffolds

**Figure 3:**
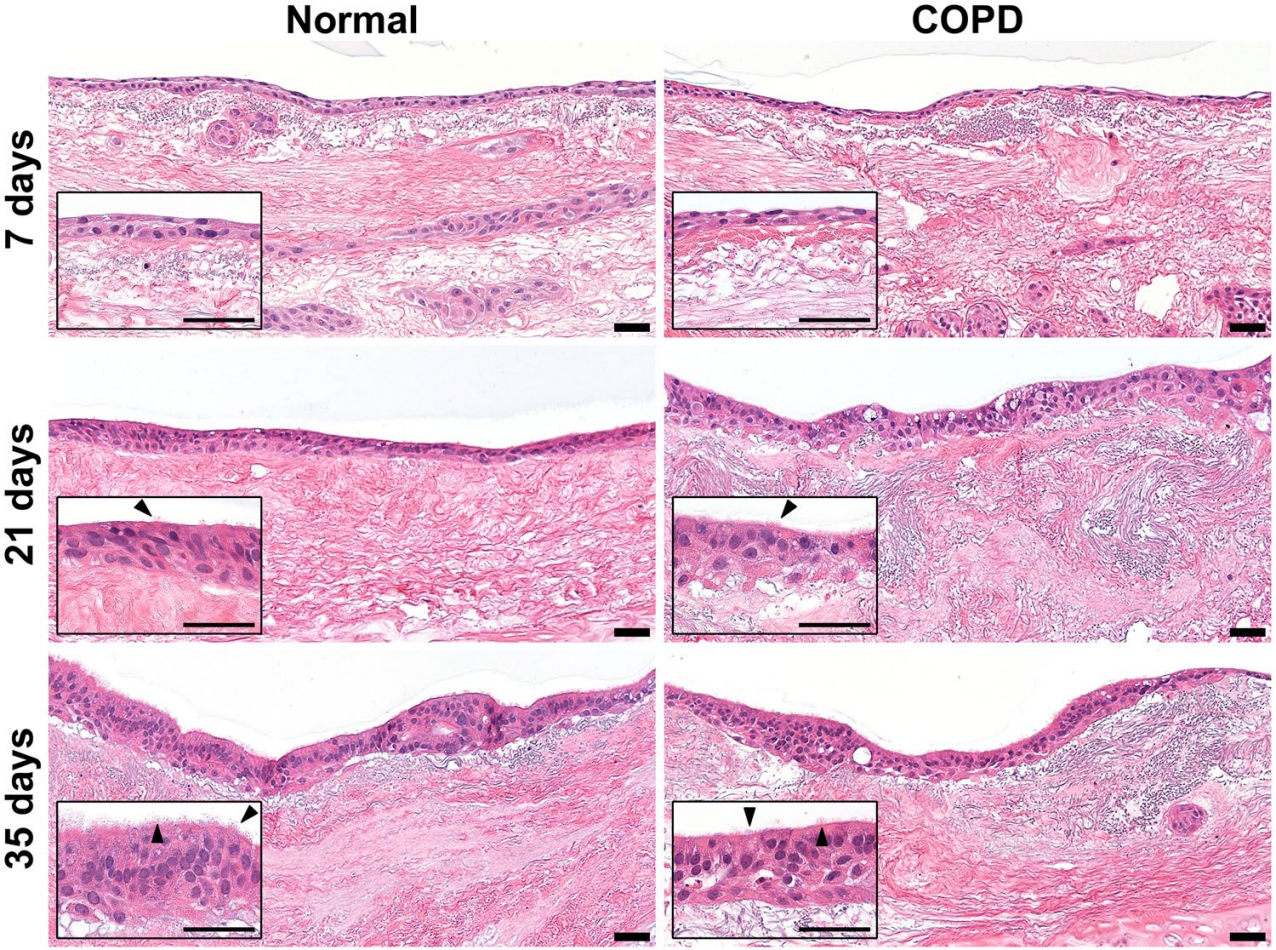
Primary normal human bronchial epithelial cells develop cilia and assume a pseudostratified morphology during differentiation on normal and COPD bronchial scaffolds. Hematoxylin & eosin staining showing the morphology of primary normal human bronchial epithelial cells differentiated on normal or COPD scaffolds for 7, 21 and 35 days. Insets show the same images at high magnification. Images are representative of n = 3. Arrows: cilia. Scale bars: 50 µm.

Primary NHBE cells were able to repopulate bronchial scaffolds from both healthy donors and COPD patients and after 7 days of differentiation a continuous layer of cells was observed on the epithelial BM and cells were also seen in the mucosa and submucosa (Fig. 3, upper panel). At this early stage of differentiation the cell layer did not yet have the morphology of a pseudostratified airway epithelium. Occasional cilia were observed on the apical side of the repopulated epithelium on both normal and COPD scaffolds after 14 days of differentiation, and after 21 days the cilia had become more prominent (Fig. 3, middle panel). At this point the cell layer had a more columnar morphology and an increased thickness compared to day 7, but the thickness was not fully consistent along the whole length of the epithelium and in some places the cells had a cuboidal shape. After 35 days of differentiation the cilia had increased in number and the epithelium had assumed a more distinct pseudostratified morphology (Fig. 3, lower panel). Occasional cells could still be seen in the mucosa and submucosa but they had decreased in number compared to earlier time points.

### Primary human bronchial epithelial cells differentiate into airway epithelium on normal and COPD bronchial scaffolds

**Figure 4:**
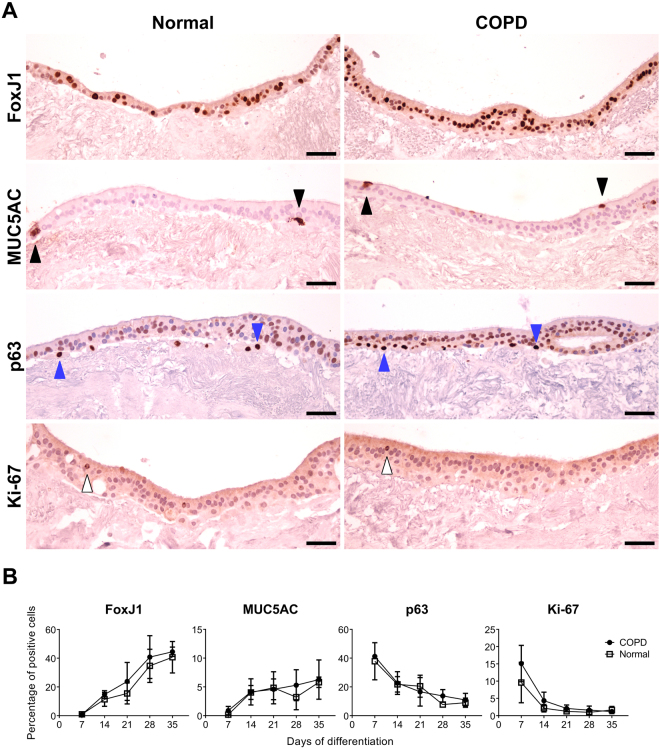
Primary normal human bronchial epithelial cells differentiate into airway epithelium on normal and COPD bronchial scaffolds. (**A**) Primary normal human bronchial epithelial cells immunostained for FoxJ1 (ciliated cells), MUC5AC (mucin 5AC) (goblet cells), p63 (basal cells) and Ki-67 (proliferation marker) after 35 days of differentiation on normal or COPD human bronchial scaffolds. Images are representative of n = 3. (**B**) Percentages of positive cells for each marker during differentiation were calculated using image analysis of immunohistochemistry results (n = 3) and counterstaining with hematoxylin allowed for counting of the total number of cells. Data were analyzed with two-way ANOVA tests using Sidak correction and graphs indicate mean and standard deviation. Black arrows: goblet cells. Blue arrows: basal cells. White arrows: Ki-67 positive cells. Scale bars: 50 µm.

Expression of FoxJ1, a marker for ciliated cells, was almost absent in the epithelium on both normal and COPD scaffolds after 7 days of differentiation (supplementary Fig. S1A) but the percentage of FoxJ1 positive (+) cells increased markedly over time (Fig. 4A,B and supplementary Fig. S1A). This increase coincided with development of cilia, confirming prominent differentiation towards a ciliated cell phenotype. Very few cells were positive for the goblet cell marker MUC5AC on both normal and COPD scaffolds after 7 days (supplementary Fig. S1B), but the proportion of MUC5AC+ cells increased over time (Fig. 4A,B and supplementary Fig. S1B). The staining intensity per cell was more pronounced at 35 days, indicating an increased MUC5AC production during later stages of differentiation. MUC5AC IHC also confirmed secretion of mucus from the repopulated cells (supplementary Fig. S2).

As expected, a large proportion of the cells were positive for the basal cell marker p63 after 7 days, but this fraction decreased over time on both normal and COPD scaffolds (Fig. 4A,B and supplementary Fig. S1C). The p63+ cells were predominantly found close to the BM. At later time points, apically located cells were observed that were positive for both p63 and FoxJ1 (supplementary Fig. S3A–D), indicating the presence of cells in a transition state between a basal cell and a ciliated cell phenotype. A kinetic pattern similar to the p63 expression was seen for the proliferation marker Ki-67 (Fig. 4A,B and supplementary Fig. S1D). The proportion of Ki-67+ cells declined dramatically between day 7 and 14 on both scaffold types and almost no positive cells were seen after 21 days, suggesting that the cells had stopped proliferating after approximately 2 weeks of differentiation.

All cells were negative for the tight junction protein ZO-1 after 7 days (supplementary Fig. S1E), but weak ZO-1 expression was observed apically after 14 days and at later time points the staining intensity had increased, demonstrating the ability of the cells to form tight junctions on both normal and COPD scaffolds.

No significant differences were seen between cells on normal and COPD scaffolds with respect to morphology or marker expression. Very few TUNEL + cells were seen on either scaffold type at any time point during differentiation (supplementary Fig. S4A,B), indicating that apoptosis is not an important factor during differentiation.

### Bronchial extracellular matrix derived from COPD patients induces differential gene expression in repopulated primary human bronchial epithelial cells

**Figure 5:**
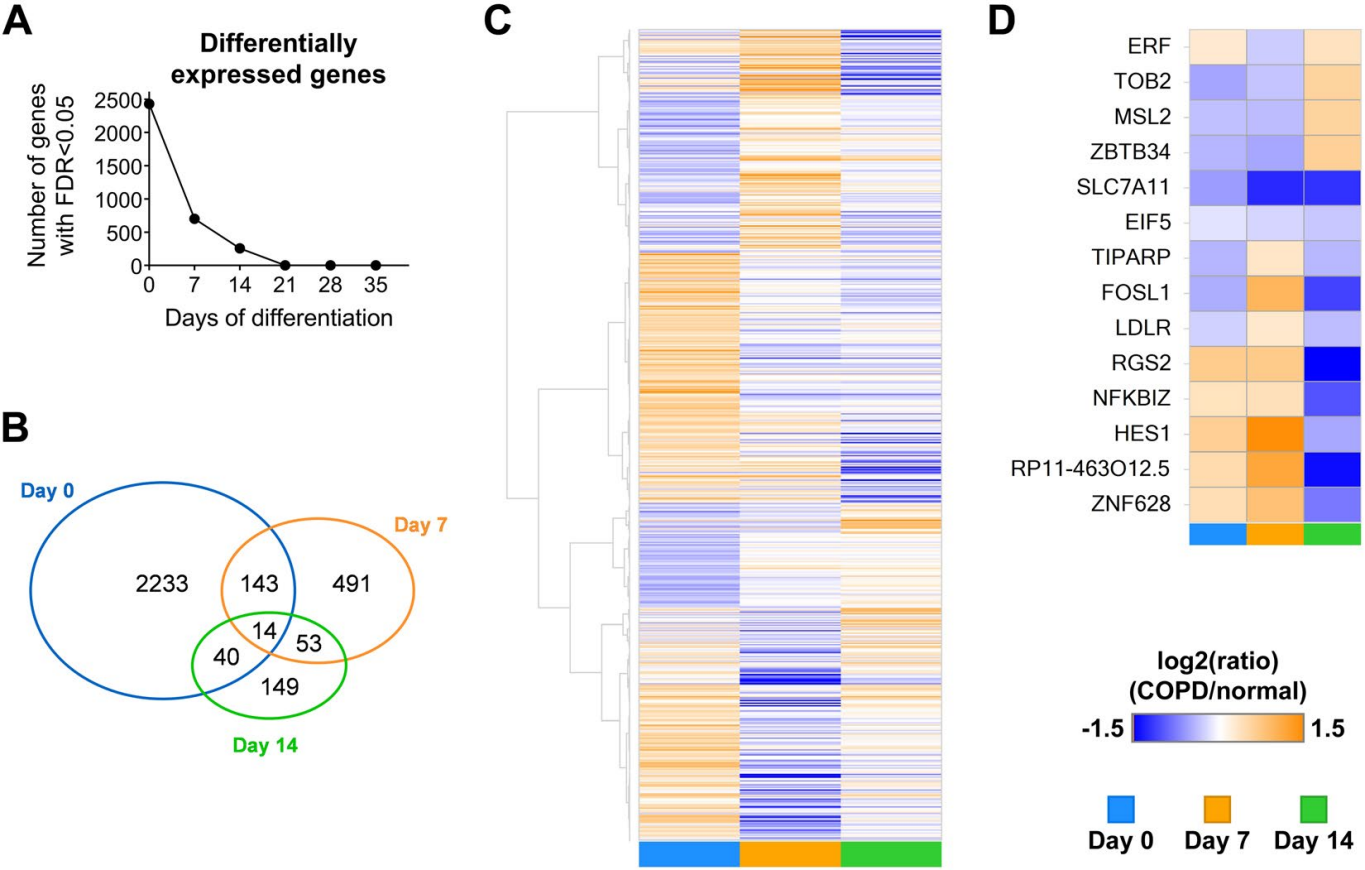
Differential gene expression in primary normal human bronchial epithelial cells differentiated on COPD compared to normal bronchial scaffolds. (**A**) The number of differentially expressed genes in cells on COPD compared to normal bronchial scaffolds at each time point during differentiation. (**B**) The number and overlap of differentially expressed genes on day 0, 7 and 14. (**C**) Heatmap showing hierarchically clustered log_2_(ratio) data where the ratio is defined as mRNA expression level in cells on COPD relative to normal scaffolds. Data are shown for all genes differentially expressed at one or more of the first three time points of differentiation. (**D**) Heatmap showing log_2_(ratio) data for the 14 genes differentially expressed at all of the first three time points. All data in this figure represent n = 3.

All samples included in the RNA-Seq study had high overall sequence quality (Phred scores, per base N content, per sequence GC content) and similar high quality profiles with respect to genomic distribution of mapped reads, gene coverage and mapping frequencies (≥97%). Global transcriptomic profiling revealed that a large number of genes were differentially expressed in NHBE cells on COPD compared to normal bronchial scaffolds and that these differences were more pronounced early during differentiation (Fig. 5A). On day 0 (when the cells had been exposed to the scaffolds for 4 days) 2430 genes were differentially expressed, but after 7 and 14 days of differentiation that number had decreased to 701 and 256, respectively (Fig. 5B). Later during differentiation (day 21–35) ≤2 genes were differentially expressed at each time point. Supplementary Table 1 shows the relative expression levels in NHBE cells on COPD relative to normal scaffolds for all genes differentially expressed at any time point.

To illustrate gene expression changes on a global scale, data for genes differentially expressed at any of the first three time points were hierarchically clustered and visualized in a heatmap (Fig. 5C). Many genes did not have a consistently higher or lower expression level on one scaffold type at all three time points. This can be seen as clusters of genes changing between orange (higher in COPD) and blue (higher in normal) color over time (Fig. 5C). Fourteen genes were differentially expressed at all of the first three time points (Fig. 5D).

**Figure 6:**
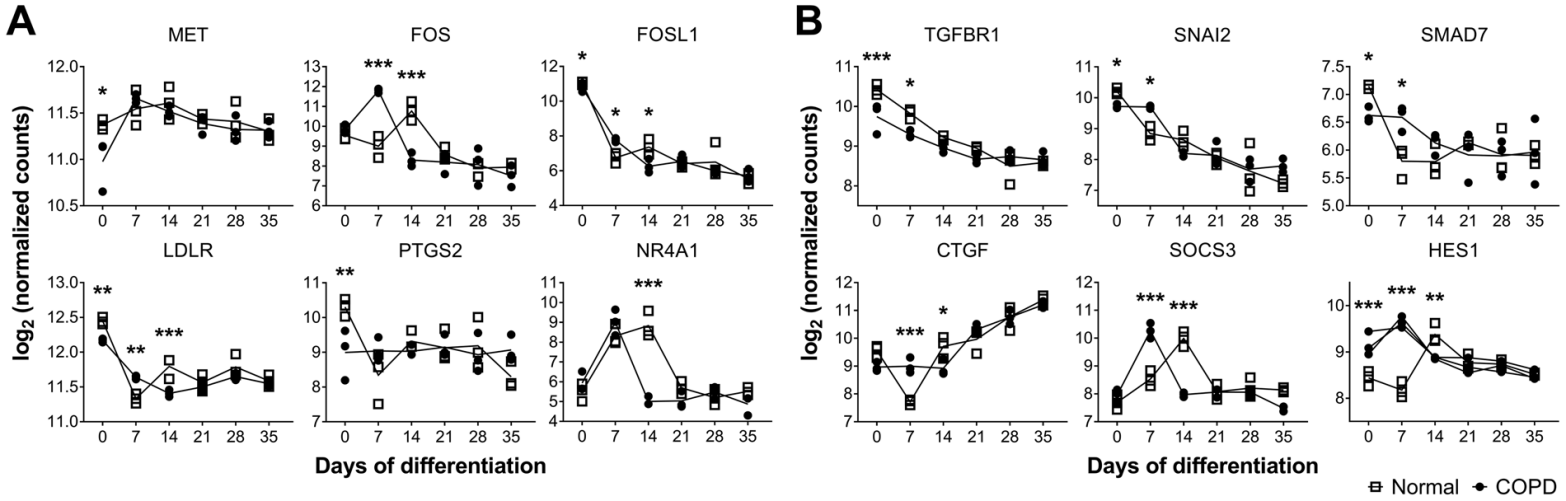
Genes regulated by HGF and TGF-β1 are differentially expressed in primary normal human bronchial epithelial cells on COPD compared to normal scaffolds. RNA-Seq data showing relative expression of genes regulated by (**A**) hepatocyte growth factor (HGF) and (**B**) transforming growth factor beta 1 (TGF-β1) in primary normal human bronchial epithelial cells during differentiation on normal (squares) or COPD (circles) bronchial scaffolds (n = 3). MET = MET proto-oncogene, receptor tyrosine kinase (HGF receptor), FOS = Fos proto-oncogene (AP-1 transcription factor subunit), FOSL1 = Fos-related antigen 1 (AP-1 transcription factor subunit), LDLR = Low density lipoprotein receptor, PTGS2 = Prostaglandin-endoperoxide synthase 2 (Cyclooxygenase 2), NR4A1 = Nuclear receptor 4A1, TGFBR1 = TGF-β receptor 1, SNAI2 = Snail family transcriptional repressor 2, SMAD7 = SMAD family member 7, CTGF = Connective tissue growth factor, SOCS3 = Suppressor of cytokine signaling 3, HES1 = Hes family bHLH transcription factor 1. The data were analyzed with DESeq2 (described in Materials and methods) using the Benjamini-Hochberg method for multiple testing correction. *FDR (False Discovery Rate) < 0.05, **FDR < 0.01, ***FDR < 0.001.

Ingenuity Pathway Analysis was used to perform upstream mediator analysis based on RNA-Seq data for genes differentially expressed at day 0, 7 and 14, respectively. Positive and negative z scores corresponded to a predicted increase or decrease in activity, respectively, in NHBE cells on COPD relative to normal bronchial scaffolds. Several mediators were predicted to have an altered activity in NHBE cells on COPD compared to normal scaffolds based on the observed expression pattern of genes downstream of those mediators (Table 2). On day 0 the gene expression pattern indicated increased activity of interferon (IFN) alpha 2, beta 1 and lambda 1 in cells on COPD scaffolds, while inhibition was predicted for HGF, TGF-β1 and estrogen receptor 1. The data were also consistent with increased activity of p53 in cells on COPD scaffolds on day 0. After 7 days of differentiation IFN alpha 2, beta 1 and lambda 1 were all predicted to have a decreased activity in cells on COPD scaffolds, while the data supported increased activity of TGF-β1. PDGF-BB, mitogen-activated protein kinase 1 (MAPK1), CD40 ligand, endothelin 1 and p53 were all predicted to have a higher activity in cells on COPD scaffolds at this time point. On day 14, all of the 10 upstream mediators with the highest absolute z score were predicted to have decreased activity in cells on COPD compared to normal scaffolds. PDGF-BB was included here, as well as several inflammatory mediators, like tumor necrosis factor alpha, IFN gamma, interleukin 1 beta and nuclear factor kappa b. As was seen on day 0, HGF was predicted to have a reduced activity on COPD scaffolds also on day 14. This was reflected in the expression pattern for genes regulated by HGF (Fig. 6A). On day 0, genes having a lower expression level in cells on COPD compared to normal scaffolds included the HGF receptor MET^19^, FOS-related antigen 1 (FOSL1)^20^, low density lipoprotein receptor (LDLR)^21^ and prostaglandin-endoperoxidase synthase 2 (PTGS2)^22^ (also known as cyclooxygenase 2). FOSL1 and LDLR had a lower expression level on COPD scaffolds also on day 14, as well as the proto-oncogene FOS^23^ and nuclear receptor 4A1 (NR4A1)^24^.

**Table 2:** Upstream mediators predicted to have a changed activity on COPD compared to normal bronchial scaffolds after 0, 7 and 14 days of differentiation.

	Upstream mediator	Predicted activity on COPD scaffolds	Activation z score	p value
**Day 0**	Estrogen receptor 1 (ESR1)	Decreased	−3.25	1.6E-08
	Hepatocyte growth factor (HGF)	Decreased	−3.18	1.8E-04
	Transforming growth factor beta 1 (TGFB1)	Decreased	−3.00	9.7E-09
	Interferon alpha 2 (IFNA2)	Increased	4.91	1.1E-04
	Interferon beta 1 (IFNB1)	Increased	4.24	8.5E-05
	Tumor protein p53 (TP53)	Increased	3.88	2.7E-10
	Interferon lambda 1 (IFNL1)	Increased	3.88	1.5E-04
	Tretinoin (all-trans retinoic acid)	Increased	3.25	2.6E-07
	Interferon alpha 1 (IFNA1)	Increased	3.11	6.6E-05
	Peroxisome proliferator activated receptor gamma (PPARG)	Increased	2.96	1.1E-04
**Day 7**	Interferon alpha 2 (IFNA2)	Decreased	−3.63	7.1E-06
	Interferon lambda 1 (IFNL1)	Decreased	−3.53	6.9E-07
	Interferon beta 1 (IFNB1)	Decreased	−3.42	8.4E-05
	Histone deacetylase (HDAC) (family)	Decreased	−3.23	1.9E-07
	Platelet-derived growth factor B (PDGF-BB)	Increased	4.74	7.4E-17
	Mitogen-activated protein kinase 1 (MAPK1)	Increased	3.94	1.2E-10
	Transforming growth factor beta 1 (TGFB1)	Increased	3.20	9.9E-11
	Tumor protein p53 (TP53)	Increased	3.15	9.4E-12
	CD40 ligand (CD40LG)	Increased	3.08	1.3E-07
	Endothelin 1 (EDN1)	Increased	3.08	2.3E-04
**Day 14**	Platelet-derived growth factor B (PDGF-BB)	Decreased	−4.76	6.7E-27
	Tumor necrosis factor alpha (TNF)	Decreased	−3.77	8.9E-16
	Nuclear factor kappa B (NF-kB) (family)	Decreased	−3.77	2.0E-08
	cAMP responsive element binding protein 1 (CREB1)	Decreased	−3.69	2.0E-13
	Calcium	Decreased	−3.67	8.2E-10
	Triggering receptor expressed on myeloid cells 1 (TREM1)	Decreased	−3.48	7.0E-10
	Coagulation factor II (thrombin) (F2)	Decreased	−3.27	6.4E-10
	Interferon gamma (IFNG)	Decreased	−3.18	2.5E-09
	Interleukin 1 beta (IL1B)	Decreased	−3.12	1.2E-20
	Hepatocyte growth factor (HGF)	Decreased	−3.12	3.1E-14

Shown are the 10 upstream mediators at each of the first three time points most likely to have a changed activity on COPD compared to normal scaffolds, based on the gene expression pattern in repopulated normal human bronchial epithelial cells (n = 3). Positive and negative activation z scores indicate increased and decreased activity, respectively, on COPD compared to normal scaffolds. The analysis was done using Ingenuity Pathway Analysis and p values were calculated with Fisher’s exact test.

Several genes known to be regulated downstream of TGF-β1 had a significantly lower expression level in cells on COPD scaffolds on day 0, including the genes for TGF-β receptor 1 (TGFBR1)^25^, Snail family transcriptional repressor 2 (SNAI2)^26^ and SMAD7^27^ (Fig. 6B). These genes were also differentially expressed on day 7, but at that time point SNAI2 and SMAD7 were more highly expressed in cells on COPD compared to normal scaffolds. Other TGF-β1 regulated genes also had a significantly higher expression level in cells on COPD scaffolds on day 7, including connective tissue growth factor (CTGF)^28^, suppressor of cytokine signaling 3 (SOCS3)^29^ and Hes family basic helix-loop-helix (bHLH) transcription factor 1 (HES1)^30^. Some genes, like FOS, NR4A1, SOCS3 and HES1 had a similar overall expression pattern in cells on COPD and normal scaffolds, but with temporal differences, reaching their peak expression earlier on COPD scaffolds.

The RNA-Seq data showed that mRNA expression aligned well with protein expression for the cellular markers used for IHC (supplementary Fig. S5), although there were some significant differences in mRNA expression between COPD and normal samples. RNA-Seq data for selected genes were validated with real-time qRT-PCR and the results were consistent between the two methods (supplementary Fig. S6).

Data for genes representing the matrisome^31^ were extracted from the RNA-Seq data and 413 out of 1068 matrisome genes were expressed across all samples. 122 of these were differentially expressed in cells on COPD compared to normal scaffolds on day 0. However, on day 7 and 14 that number had decreased to 27 and 22, respectively, and on day 21–35 no more than one gene was differentially expressed at each time point. This demonstrates a converging expression pattern also for genes encoding ECM proteins (supplementary Fig. S7A,B).

## Discussion

We demonstrate that bronchial ECM derived from COPD patients induces altered gene expression in differentiating NHBE cells at an early stage of differentiation. The differential gene expression pattern indicated altered activity of upstream mediators that regulate pathways involved in regeneration (HGF) and remodeling (TGF-β1), but also apoptosis (p53), vascularization (PDGF-BB, endothelin 1) and inflammation (IFNs), which are all relevant to COPD pathophysiology. These findings are based on a new *ex vivo* model we developed that offers a promising experimental platform for studies on interactions between bronchial ECM and primary bronchial epithelial cells.

In the present study, HGF was predicted to have a lower activity in NHBE cells on COPD compared to normal bronchial scaffolds in the upstream mediator analysis (Table 2). This mediator has been shown to be downregulated in epithelial lining fluid of peripheral airways in COPD patients^32^. HGF is also involved in epithelial regeneration by promoting proliferation and survival of airway epithelial cells^33,34^. It induces bronchioalveolar-like branching from colonies of bronchial epithelial cells^35^ as well as branching of airways in the developing lung^36^. HGF is known to bind to molecules in the ECM and some of those interactions have been shown to depend on the fine structure of certain GAGs^37^, which may therefore affect HGF retention in the ECM. In addition, fibroblasts derived from emphysematous lungs have an impaired HGF production^38^. Structural alterations of ECM molecules, like GAGs, and/or decreased HGF production from fibroblasts may therefore lead to a lower amount of HGF in the bronchial ECM of COPD patients, which could explain the altered expression of HGF regulated genes in the RNA-Seq data.

Moreover, the differential gene expression profile also indicated dysregulated activation of genes downstream of TGF-β1 in NHBE cells on COPD compared to normal scaffolds (Table 2). On day 7, several genes known to be induced by TGF-β1^26–30^ were more highly expressed in cells on COPD scaffolds, indicating enhanced TGF-β1 signaling (Fig. 6B). On day 0, genes like SNAI2 and SMAD7 were more highly expressed in NHBE cells on normal compared to COPD scaffolds, but the expression decreased towards day 7 on normal scaffolds. However, in cells on COPD scaffolds the expression remained at the same level and decreased only after day 7, indicating a maintained activation of the TGF-β1 pathway in the presence of COPD scaffolds. TGF-β1 is a multifunctional growth factor involved in processes such as lung development^39^, tissue repair and fibrosis^40,41^. It has been found to be associated with clinical severity and airflow limitation in COPD patients^42^ and also plays a role in regulating ECM production^43^ as well as modulating the fine structure of GAGs^44^. Interestingly, both bronchial epithelial cells and fibroblasts isolated from COPD patients have been shown to have an elevated TGF-β1 production^45,46^. The gene expression pattern in our RNA-Seq data suggests that alterations in the bronchial ECM may contribute to dysregulation of TGF-β1 signaling in the airway epithelium of COPD patients. This could be a reflection of disease-related remodeling of the ECM. Chen *et al*.^8^ showed that heparan sulfate mediates binding between latent TGF-β-binding protein 1 and fibronectin, thereby indirectly regulating the availability of latent TGF-β1 in the ECM. Heparan sulfate proteoglycans such as perlecan make up a vital part of the ECM and possible alterations in the production or structure of these molecules could affect retention of TGF-β1 in the ECM.

PDGF-BB and MAPK1, more known as extracellular-signal regulated kinase 2 (ERK2), were also predicted to have an altered activity in cells on COPD compared to normal scaffolds (Table 2). PDGF-BB is a growth factor implicated in pulmonary vascular remodeling^47^ and it is overexpressed in arteries of patients with pulmonary arterial hypertension^48^, which is a common comorbidity in COPD patients^49^. Furthermore, ERK2 is a part of the signal transduction cascade downstream of the PDGF receptors^50^. Our RNA-Seq data indicate that bronchial ECM from COPD patients may contribute to dysregulated signaling along the PDGF-BB/ERK2 axis, which could reflect processes of vascular remodeling in COPD patients.

Taken together, our gene expression data imply that bronchial ECM in COPD patients have compositional and/or architectural changes that induce differential gene expression in NHBE cells. These results, and the roles of HGF, TGF-β1 and PDGF-BB in regeneration and remodeling, raise the possibility that bronchial ECM may contribute to the impaired regenerative phenotype in COPD lungs.

Airway epithelial cells have been cultured on substrates like collagen I^51^ and Matrigel^52^, but such environments do not provide the cells with the natural anchoring points and biochemical cues of their tissue-specific ECM. The new *ex vivo* model presented here allows the cells to interact with the more physiological microenvironment offered by a native ECM scaffold. To our knowledge, this is the first time that primary human bronchial epithelial cells have been shown to be able to reach such a high degree of differentiation on airway scaffolds derived from patients with respiratory disease. Human airway epithelial cells have previously been used to repopulate human lung scaffolds with various experimental setups. Wagner *et al*.^53^ used excised segments of decellularized whole lungs from healthy donors and emphysematous COPD patients for repopulation with human bronchial epithelial cells, which remained viable for up to 21 days after inoculation into normal lung scaffolds but only for 7 days in COPD lung scaffolds. The cells were found lining alveolar septa and airways but did not assume a pseudostratified morphology. Marker expression demonstrated a rapid decrease in proliferation and a low degree of apoptosis, which is in agreement with our results. Gilpin *et al*.^54^ repopulated scaffolds from whole lobes of human lungs with a population of human airway basal cells in a bioreactor setting, and confirmed expression of FOXJ1, p63 and Ki-67 mRNA after 7 days of tissue culture, but they did not investigate later time points. The nature of our model allows the epithelial cells to differentiate close to an air-liquid interface after repopulation, which may partially explain why we see more pronounced differentiation. However, another reason for the different outcomes between previous studies and the present study could be differences in ECM composition in bronchial airways compared to distal airways and whole lungs.

Finally, an important observation in the RNA-Seq data is the fading differential effect COPD bronchial scaffolds have on gene expression in NHBE cells as differentiation progresses (Fig. 5A,B). Even though diseased and normal ECM will affect cells differently, the repopulated NHBE cells will also have an impact on their new environment by their production of ECM molecules. The converging gene expression we see at later time points could be a consequence of the inherent capacity of these cells to produce normal ECM, which may over time neutralize the disease-related biochemical signature of the COPD scaffolds. This is supported by our RNA-Seq data, which show a converging expression pattern also for matrisome^31^ genes (supplementary Fig. S7A,B). One also has to consider that all cells in this study, regardless of scaffold type, differentiated in the same type of medium, which might have had a normalizing effect on gene expression over time. No obvious phenotypic difference was seen between NHBE cells on COPD and normal scaffolds with respect to marker expression (Fig. 4B and supplementary Fig. S1) or morphology (Fig. 3), which suggests that the influence of the diseased bronchial ECM on gene expression in NHBE cells is more functional than structural. We used primary NHBE cells from a single donor as responder cells to compare the gene expression patterns after exposure to bronchial ECM from multiple COPD patients and controls. The use of NHBE cells from a single donor was a way to standardize the experiment, and we could not use cells from different donors as responder cells due to limitations of the tissue supply from patients and controls.

This model provides a new platform for studies on epithelial cell-ECM interactions. Our findings initiate further studies on identification of factors in the ECM causing pathological changes in the airways of COPD patients. Furthermore, it is also of interest how normal bronchial ECM modulates the phenotype of bronchial epithelial cells derived from COPD patients, and whether a normal bronchial ECM would revert a diseased epithelial phenotype.

## Electronic supplementary material

Supplementary Figures

Supplementary table 1

## References

[1] McDonough JE (2011). Small-airway obstruction and emphysema in chronic obstructive pulmonary disease. The New England journal of medicine.

[2] Vestbo J (2013). Global strategy for the diagnosis, management, and prevention of chronic obstructive pulmonary disease: GOLD executive summary. American journal of respiratory and critical care medicine.

[3] Mathers CD, Loncar D (2006). Projections of global mortality and burden of disease from 2002 to 2030. PLoS medicine.

[4] Rigden HM (2016). Squamous Metaplasia Is Increased in the Bronchial Epithelium of Smokers with Chronic Obstructive Pulmonary Disease. PloS one.

[5] Innes AL (2006). Epithelial mucin stores are increased in the large airways of smokers with airflow obstruction. Chest.

[6] Shaykhiev R (2011). Cigarette smoking reprograms apical junctional complex molecular architecture in the human airway epithelium *in vivo*. Cellular and molecular life sciences: CMLS.

[7] Kresse H, Schonherr E (2001). Proteoglycans of the extracellular matrix and growth control. Journal of cellular physiology.

[8] Chen Q (2007). Potential role for heparan sulfate proteoglycans in regulation of transforming growth factor-beta (TGF-beta) by modulating assembly of latent TGF-beta-binding protein-1. The Journal of biological chemistry.

[9] Handel TM, Johnson Z, Crown SE, Lau EK, Proudfoot AE (2005). Regulation of protein function by glycosaminoglycans–as exemplified by chemokines. Annual review of biochemistry.

[10] Annoni R (2012). Extracellular matrix composition in COPD. The European respiratory journal.

[11] Kranenburg AR (2006). Enhanced bronchial expression of extracellular matrix proteins in chronic obstructive pulmonary disease. American journal of clinical pathology.

[12] Harju T (2010). Variability in the precursor proteins of collagen I and III in different stages of COPD. Respiratory research.

[13] Westergren-Thorsson G (2017). Increased deposition of glycosaminoglycans and altered structure of heparan sulfate in idiopathic pulmonary fibrosis. The international journal of biochemistry & cell biology.

[14] Kim, D., Langmead, B. & Salzberg, S. L. HISAT: a fast spliced aligner with low memory requirements. **12**, 357–360, 10.1038/nmeth.3317 (2015). 10.1038/nmeth.3317 PMC4655817 25751142

[15] Liao Y, Smyth GK, Shi W (2014). featureCounts: an efficient general purpose program for assigning sequence reads to genomic features. Bioinformatics (Oxford, England).

[16] Love MI, Huber W, Anders S (2014). Moderated estimation of fold change and dispersion for RNA-seq data with DESeq. 2. Genome biology.

[17] Kramer A, Green J, Pollard J, Tugendreich S (2014). Causal analysis approaches in Ingenuity Pathway Analysis. Bioinformatics (Oxford, England).

[18] Crapo PM, Gilbert TW, Badylak SF (2011). An overview of tissue and whole organ decellularization processes. Biomaterials.

[19] McGill GG, Haq R, Nishimura EK, Fisher DE (2006). c-Met expression is regulated by Mitf in the melanocyte lineage. The Journal of biological chemistry.

[20] Ramos-Nino ME (2008). HGF mediates cell proliferation of human mesothelioma cells through a PI3K/MEK5/Fra-1 pathway. American journal of respiratory cell and molecular biology.

[21] Dhawan P, Bell A, Kumar A, Golden C, Mehta KD (1999). Critical role of p42/44(MAPK) activation in anisomycin and hepatocyte growth factor-induced LDL receptor expression: activation of Raf-1/Mek-1/p42/44(MAPK) cascade alone is sufficient to induce LDL receptor expression. Journal of lipid research.

[22] Siegfried JM, Gubish CT, Rothstein ME, Queiroz de Oliveira PE, Stabile LP (2007). Signaling pathways involved in cyclooxygenase-2 induction by hepatocyte growth factor in non small-cell lung cancer. Molecular pharmacology.

[23] Takeuchi K (2001). Signaling pathways leading to transcription and translation cooperatively regulate the transient increase in expression of c-Fos protein. The Journal of biological chemistry.

[24] Gerritsen ME, Tomlinson JE, Zlot C, Ziman M, Hwang S (2003). Using gene expression profiling to identify the molecular basis of the synergistic actions of hepatocyte growth factor and vascular endothelial growth factor in human endothelial cells. British journal of pharmacology.

[25] Li HX (2010). Kruppel-like factor 4 promotes differentiation by transforming growth factor-beta receptor-mediated Smad and p38 MAPK signaling in vascular smooth muscle cells. The Journal of biological chemistry.

[26] Liu YN (2012). Critical and reciprocal regulation of KLF4 and SLUG in transforming growth factor beta-initiated prostate cancer epithelial-mesenchymal transition. Molecular and cellular biology.

[27] Saito A (2013). An integrated expression profiling reveals target genes of TGF-beta and TNF-alpha possibly mediated by microRNAs in lung cancer cells. PloS one.

[28] Sandbo N (2013). Control of myofibroblast differentiation by microtubule dynamics through a regulated localization of mDia2. The Journal of biological chemistry.

[29] Fox SW, Haque SJ, Lovibond AC, Chambers TJ (2003). The possible role of TGF-beta-induced suppressors of cytokine signaling expression in osteoclast/macrophage lineage commitment *in vitro*. Journal of immunology (Baltimore, Md.: 1950).

[30] Kennard S, Liu H, Lilly B (2008). Transforming growth factor-beta (TGF- 1) down-regulates Notch3 in fibroblasts to promote smooth muscle gene expression. The Journal of biological chemistry.

[31] Naba, A. *et al*. The matrisome: *in silico* definition and *in vivo* characterization by proteomics of normal and tumor extracellular matrices. Molecular & cellular proteomics: MCP **11**, M111.014647, 10.1074/mcp.M111.014647 (2012). 10.1074/mcp.M111.014647 PMC3322572 22159717

[32] Kanazawa H, Tochino Y, Asai K, Hirata K (2014). Simultaneous assessment of hepatocyte growth factor and vascular endothelial growth factor in epithelial lining fluid from patients with COPD. Chest.

[33] Cahill EF, Kennelly H, Carty F, Mahon BP, English K (2016). Hepatocyte Growth Factor Is Required for Mesenchymal Stromal Cell Protection Against Bleomycin-Induced Pulmonary Fibrosis. Stem cells translational medicine.

[34] Calvi C (2013). Hepatocyte growth factor, a determinant of airspace homeostasis in the murine lung. PLoS genetics.

[35] Kato T, Oka K, Nakamura T, Ito A (2015). Bronchioalveolar morphogenesis of human bronchial epithelial cells depending upon hepatocyte growth factor. Journal of cellular and molecular medicine.

[36] Ohmichi H, Koshimizu U, Matsumoto K, Nakamura T (1998). Hepatocyte growth factor (HGF) acts as a mesenchyme-derived morphogenic factor during fetal lung development. Development (Cambridge, England).

[37] Thelin MA (2012). Dermatan sulfate is involved in the tumorigenic properties of esophagus squamous cell carcinoma. Cancer research.

[38] Plantier L (2005). Defect of hepatocyte growth factor production by fibroblasts in human pulmonary emphysema. American journal of physiology. Lung cellular and molecular physiology.

[39] Morty RE, Konigshoff M, Eickelberg O (2009). Transforming growth factor-beta signaling across ages: from distorted lung development to chronic obstructive pulmonary disease. Proceedings of the American Thoracic Society.

[40] Westergren-Thorsson G (1993). Altered expression of small proteoglycans, collagen, and transforming growth factor-beta 1 in developing bleomycin-induced pulmonary fibrosis in rats. J Clin Invest.

[41] Fernandez IE, Eickelberg O (2012). The impact of TGF-beta on lung fibrosis: from targeting to biomarkers. Proceedings of the American Thoracic Society.

[42] Chiang CH, Chuang CH, Liu SL (2014). Transforming growth factor-beta1 and tumor necrosis factor-alpha are associated with clinical severity and airflow limitation of COPD in an additive manner. Lung.

[43] Westergren-Thorsson G, Antonsson P, Malmstrom A, Heinegard D, Oldberg A (1991). The synthesis of a family of structurally related proteoglycans in fibroblasts is differently regulated by TFG-beta. Matrix (Stuttgart, Germany).

[44] Tiedemann K (2005). Regulation of the chondroitin/dermatan fine structure by transforming growth factor-beta1 through effects on polymer-modifying enzymes. Glycobiology.

[45] Gohy ST (2014). Polymeric immunoglobulin receptor down-regulation in chronic obstructive pulmonary disease. Persistence in the cultured epithelium and role of transforming growth factor-beta. American journal of respiratory and critical care medicine.

[46] Togo S (2008). Lung fibroblast repair functions in patients with chronic obstructive pulmonary disease are altered by multiple mechanisms. American journal of respiratory and critical care medicine.

[47] Liang S (2017). PDGF-BB/KLF4/VEGF Signaling Axis in Pulmonary Artery Endothelial Cell Angiogenesis. Cellular physiology and biochemistry: international journal of experimental cellular physiology, biochemistry, and pharmacology.

[48] Perros F (2008). Platelet-derived growth factor expression and function in idiopathic pulmonary arterial hypertension. American journal of respiratory and critical care medicine.

[49] Chaouat A, Naeije R, Weitzenblum E (2008). Pulmonary hypertension in COPD. The European respiratory journal.

[50] Noskovicova N, Petrek M, Eickelberg O, Heinzelmann K (2015). Platelet-derived growth factor signaling in the lung. From lung development and disease to clinical studies. American journal of respiratory cell and molecular biology.

[51] Pageau SC, Sazonova OV, Wong JY, Soto AM, Sonnenschein C (2011). The effect of stromal components on the modulation of the phenotype of human bronchial epithelial cells in 3D culture. Biomaterials.

[52] Fessart D, Begueret H, Delom F (2013). Three-dimensional culture model to distinguish normal from malignant human bronchial epithelial cells. The European respiratory journal.

[53] Wagner DE (2014). Comparative decellularization and recellularization of normal versus emphysematous human lungs. Biomaterials.

[54] Gilpin SE (2016). Regenerative potential of human airway stem cells in lung epithelial engineering. Biomaterials.

