# Supplementary material for: Bronchial extracellular matrix from COPD patients induces altered gene expression in repopulated primary human bronchial epithelial cells
Source: Sci Rep. 2018 Feb 22;8:3502. doi: 10.1038/s41598-018-21727-w (PMC5823945; doi:10.1038/s41598-018-21727-w)
Supplement: Supplementary file 1 — Supplementary Figures [file 41598_2018_21727_MOESM1_ESM.doc]

**Bronchial extracellular matrix from COPD patients induces altered gene expression in repopulated primary human bronchial epithelial cells**

Ulf Hedström^1,2^, Oskar Hallgren^2,3^, Lisa Öberg^4^, Amy DeMicco^1^, Outi Vaarala^4^, Gunilla Westergren-Thorsson^2†^, Xiaohong Zhou^1†*^

^1^ Bioscience Regeneration Department, Respiratory, Inflammation and Autoimmunity, IMED Biotech Unit, AstraZeneca, Gothenburg, Sweden

^2^ Division of Lung Biology, Department of Experimental Medical Science, Lund University, Lund, Sweden

^3^ Division of Respiratory Medicine and Allergology, Department of Clinical Sciences, Lund University, Lund, Sweden

^4^ Bioscience Immunity Department, Respiratory, Inflammation and Autoimmunity, IMED Biotech Unit, AstraZeneca, Gothenburg, Sweden

^†^The authors contributed equally to the work

^*^Correspondence to: Xiaohong Zhou, AstraZeneca R&D Gothenburg, Pepparedsleden 1,

S-43183 Mölndal, Sweden. E-mail address: Xiao-Hong.Zhou@astrazeneca.com

**Supplementary figures**

Figure S1A


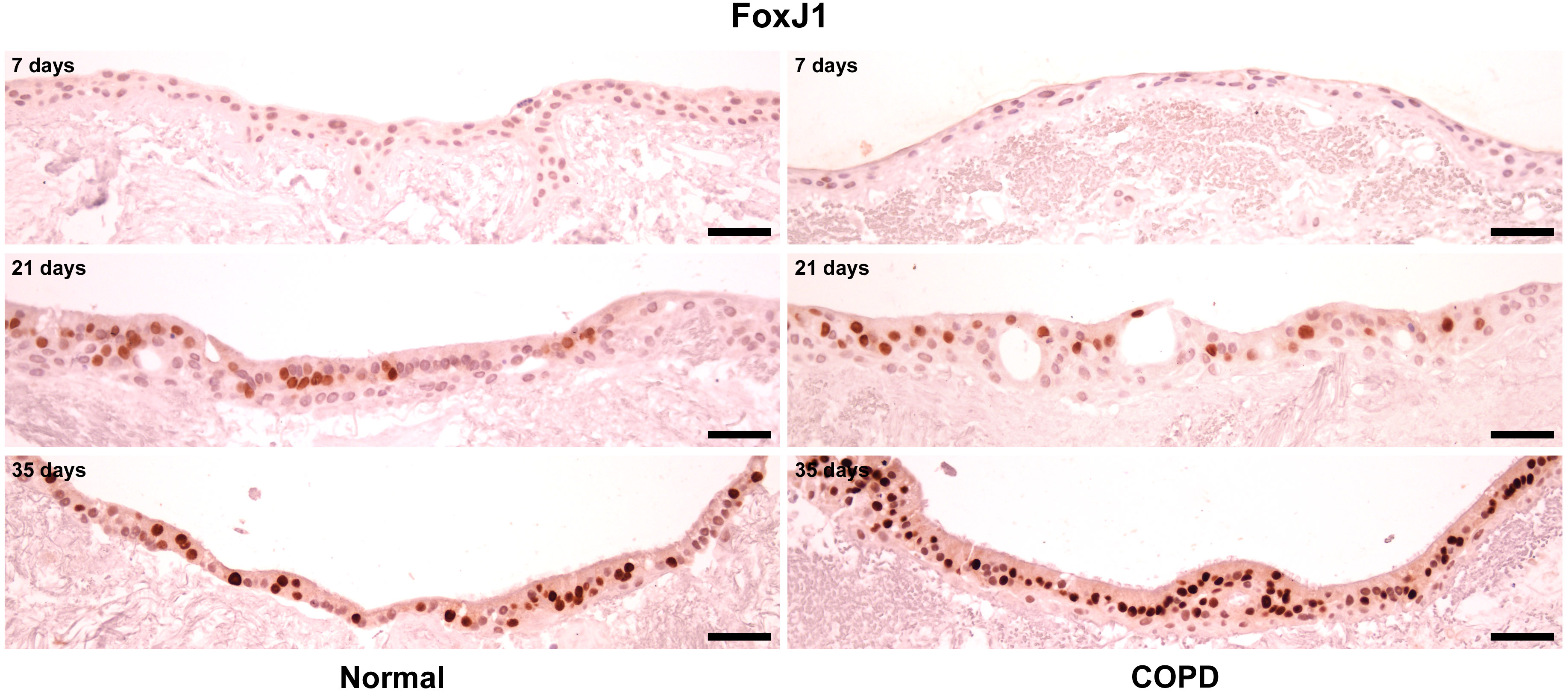


Figure S1B


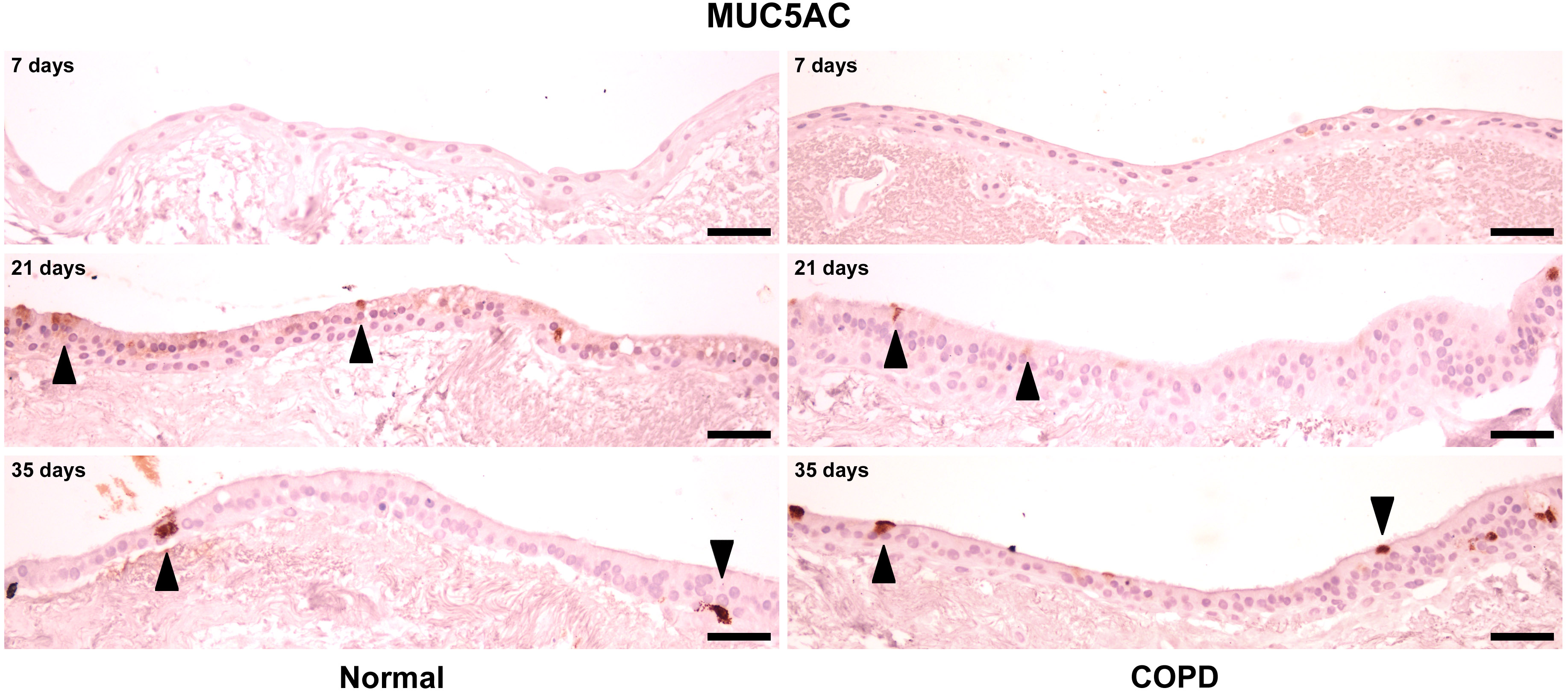


Figure S1C


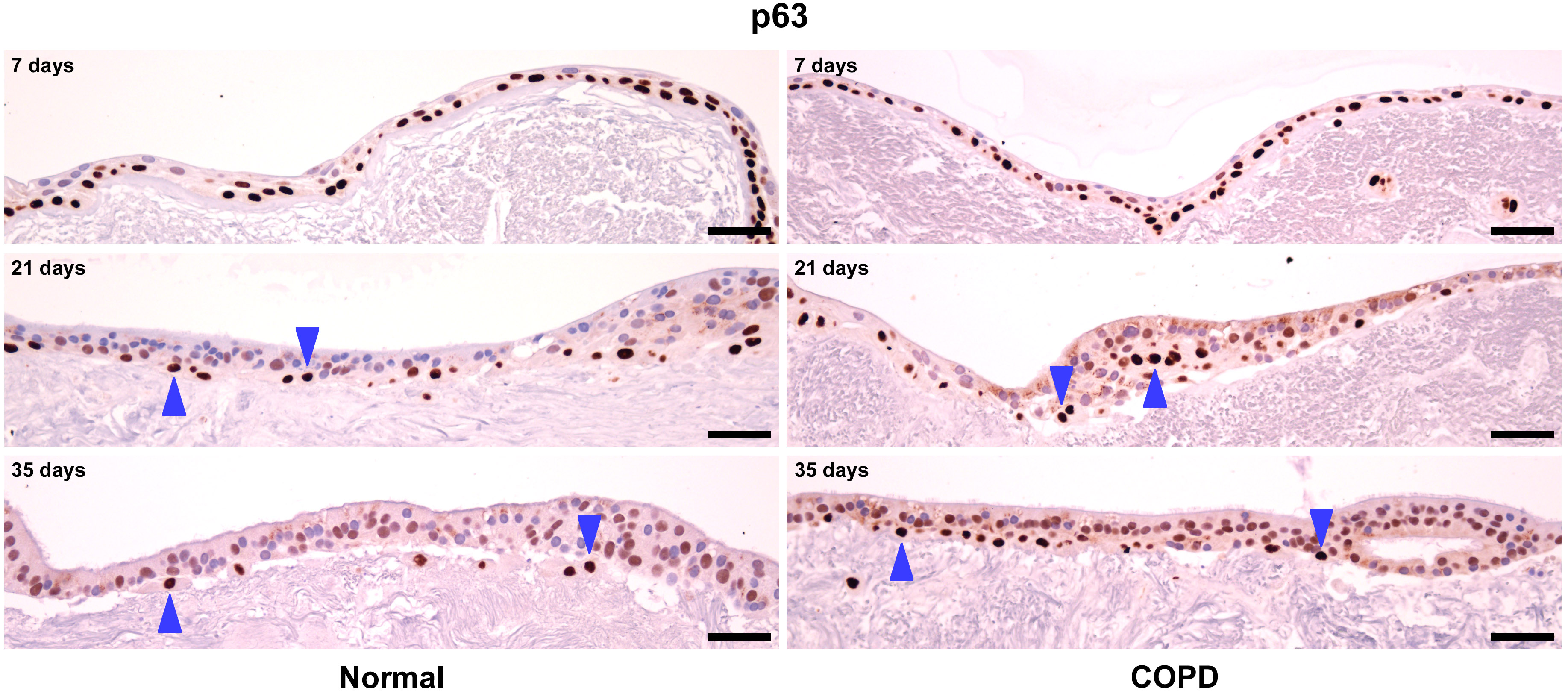


Figure S1D


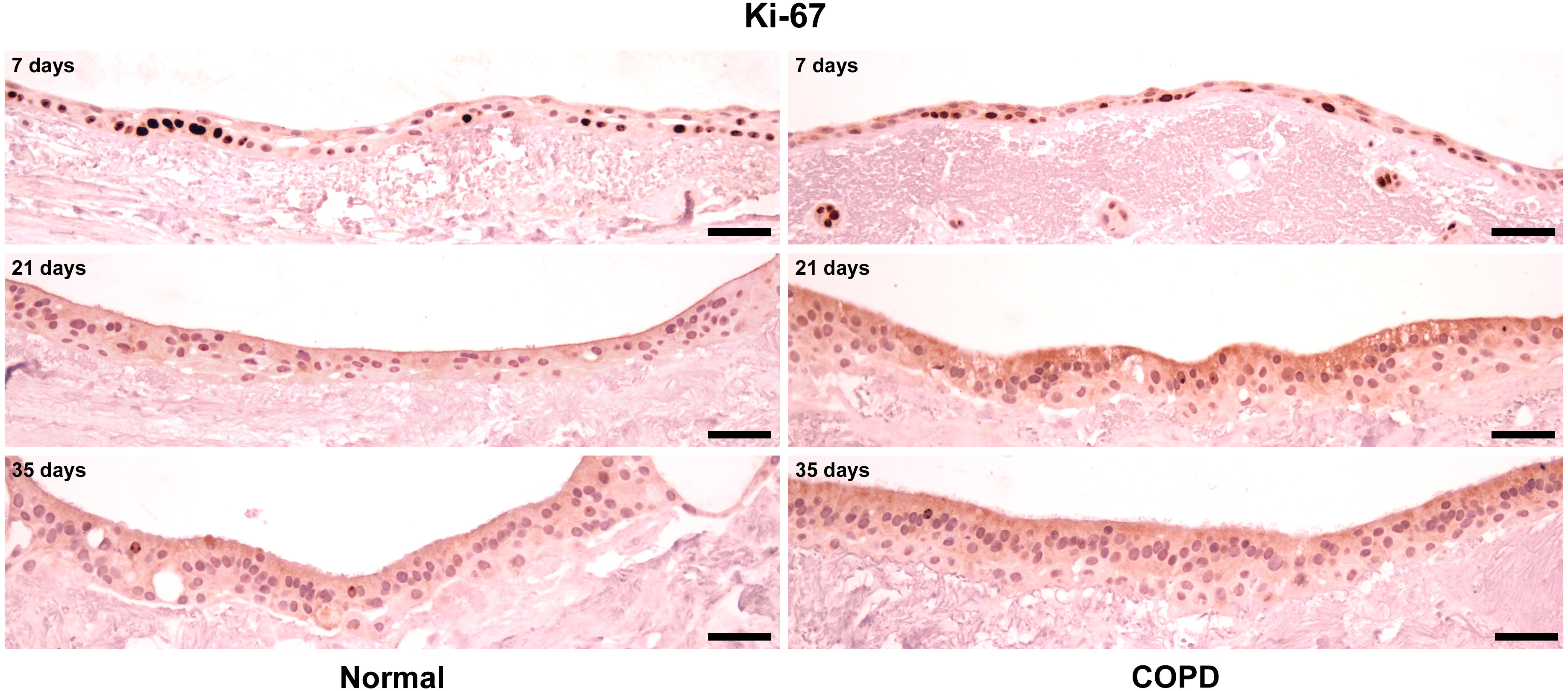


Figure S1E


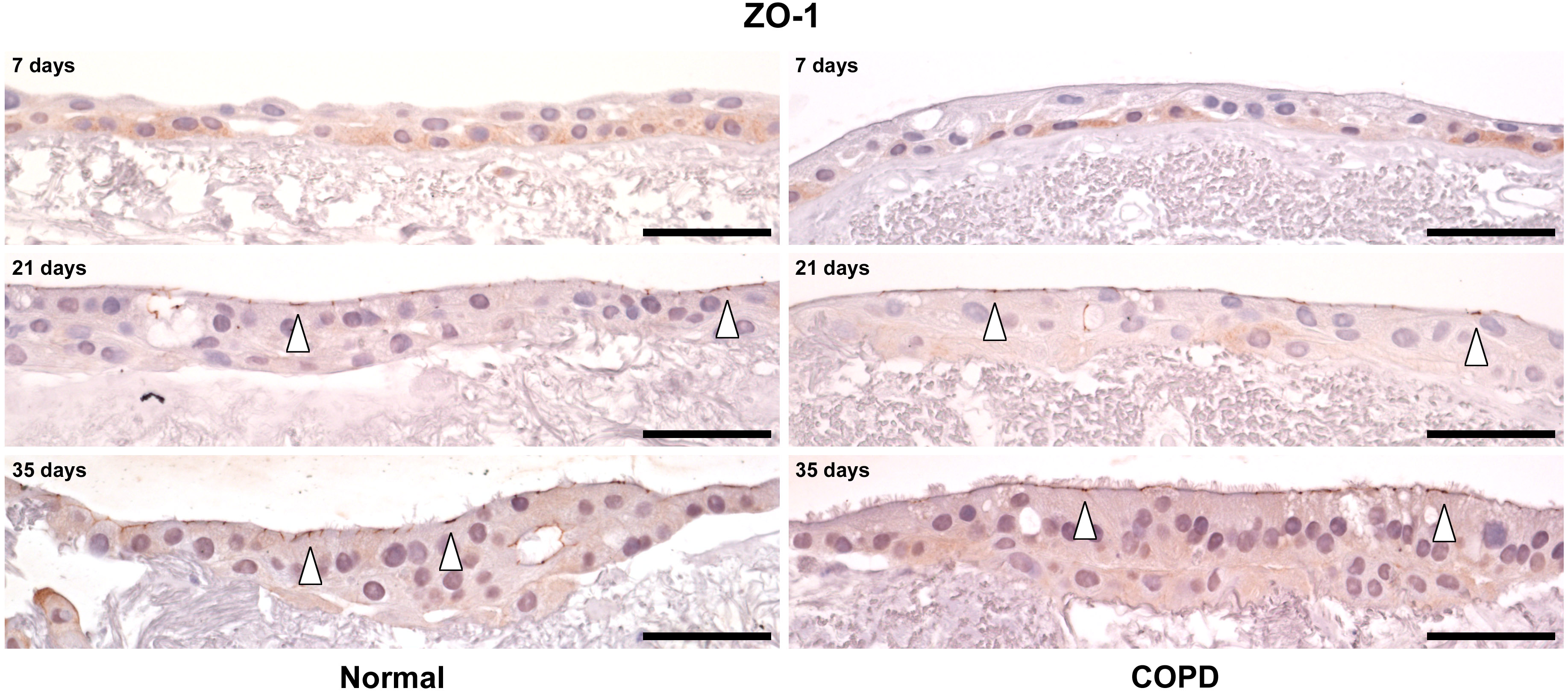


**Figure S1. Immunohistochemistry against epithelial cell markers and Ki-67 after 7, 21 and 35 days of differentiation on normal or COPD bronchial scaffolds.** Primary normal human bronchial epithelial cells immunostained for **(A)** FoxJ1 (ciliated cells), **(B)** MUC5AC (mucin 5AC) (goblet cells), **(C)** p63 (basal cells), **(D)** Ki-67 (proliferation marker) and **(E)** ZO-1 (tight junctions) after 7, 21 and 35 days of differentiation on normal or COPD bronchial scaffolds. All images are representative of n=3. Black arrows: goblet cells. Blue arrows: basal cells. White arrows: tight junctions. Scale bars: 50 µm.

Figure S2


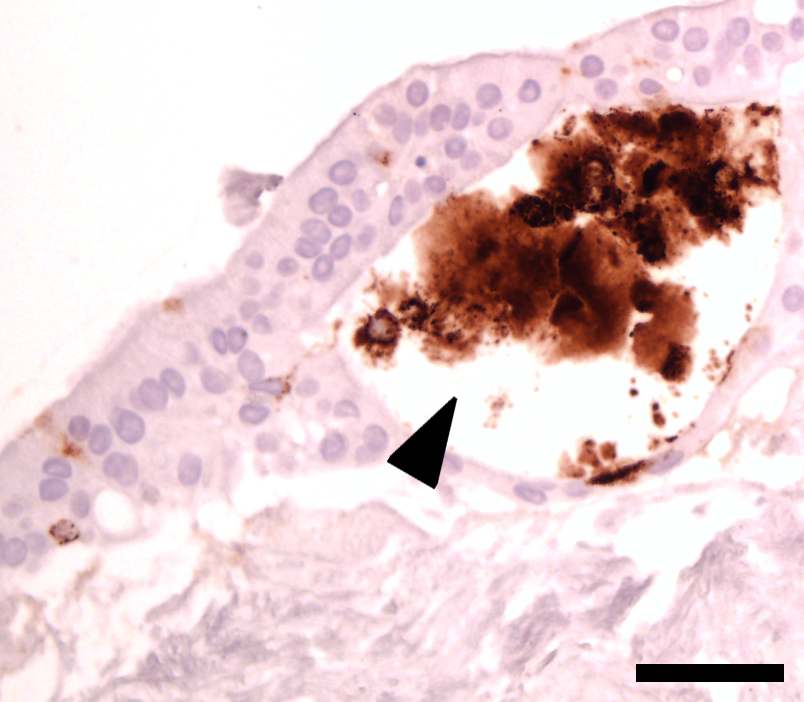


**Figure S2. Mucus production from primary normal human bronchial epithelial cells after 35 days of differentiation on bronchial scaffolds.** Mucin 5AC immunohistochemistry demonstrating mucus production (arrow). The image is representative of n=3. Scale bar: 50 µm.

Figure S3


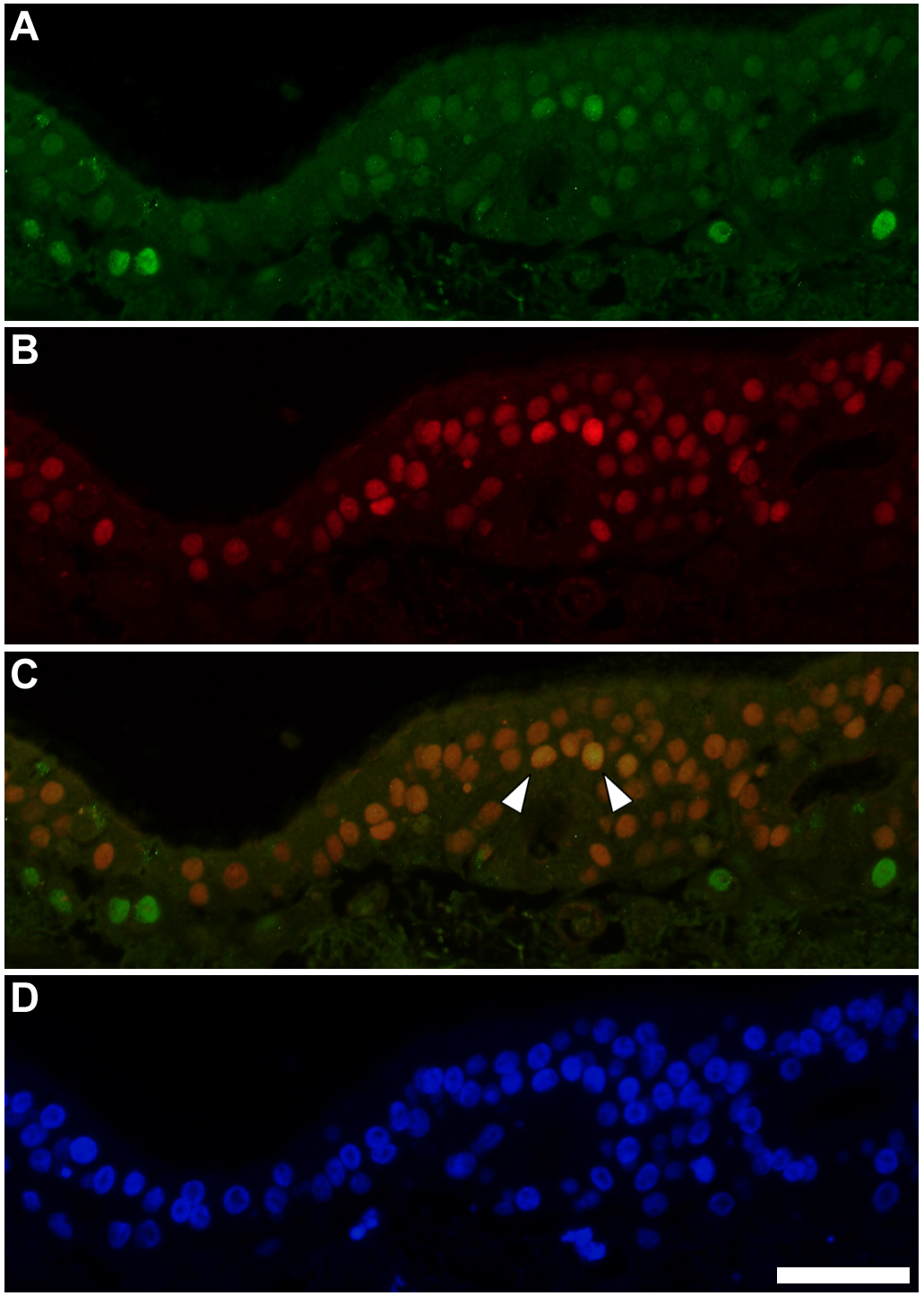


**Figure S3. Coexpression of p63 and FoxJ1 in primary normal human bronchial epithelial cells on normal bronchial scaffold after 35 days of differentiation.** Double staining by immunohistochemistry against **(A)** p63 (green) and **(B)** FoxJ1 (red). **(C)** Merged image shows weakly p63 positive cells that are also FoxJ1 positive (arrows), suggesting that basal cells differentiate towards a ciliated cell phenotype in the repopulated epithelium. **(D)** Nuclei were stained with DAPI (blue). Scale bar: 50 µm.

Figure S4A


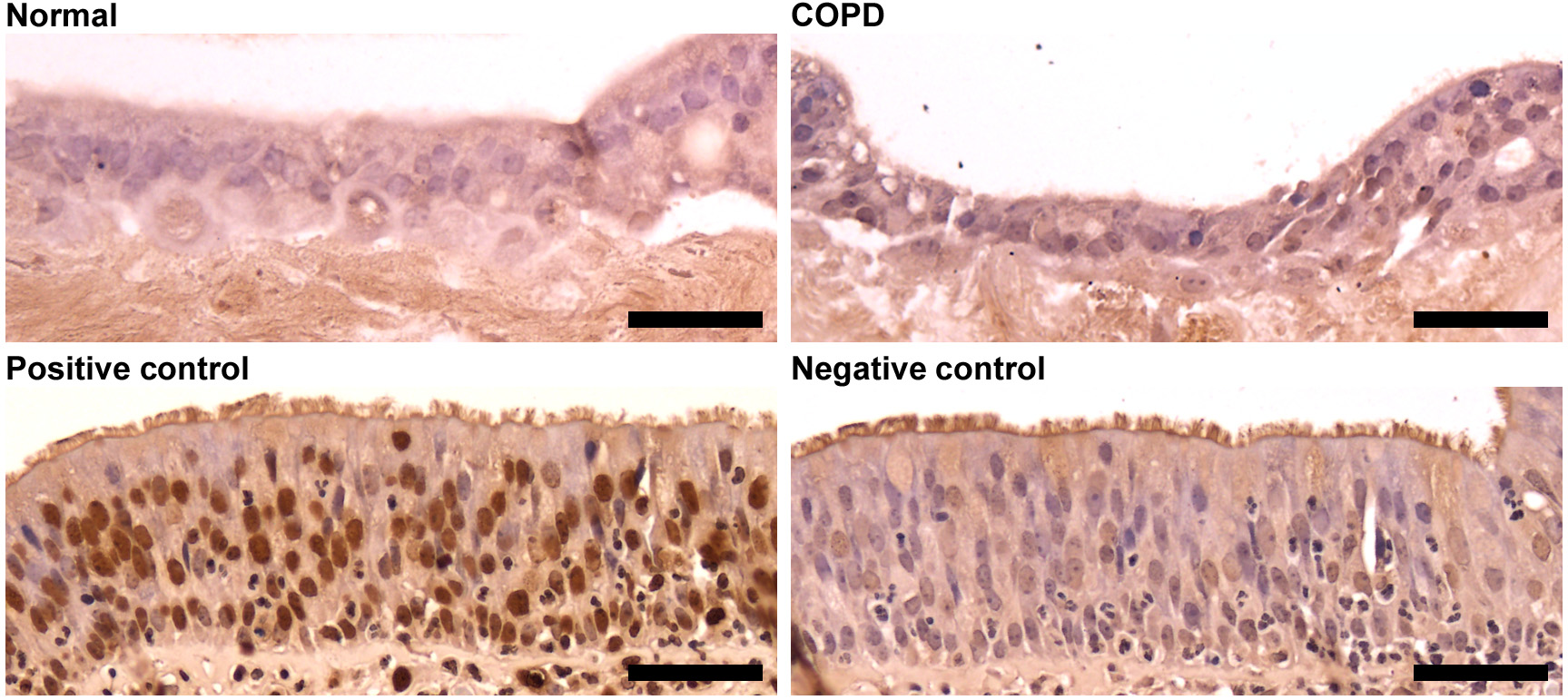


**Figure S4A. TUNEL staining of primary normal human bronchial epithelial cells differentiated for 35 days on normal or COPD bronchial scaffolds.** Native bronchial tissue sections were used as controls. Positive control tissue was pre-treated with deoxyribonuclease I. For the negative control tissue the TdT enzyme catalyzing the TUNEL reaction was omitted. TUNEL=TdT-mediated dUTP Nick-End Labeling. TdT= Terminal Deoxynucleotidyl Transferase. Images are representative of n=3. Scale bars: 50 µm.

Figure S4B


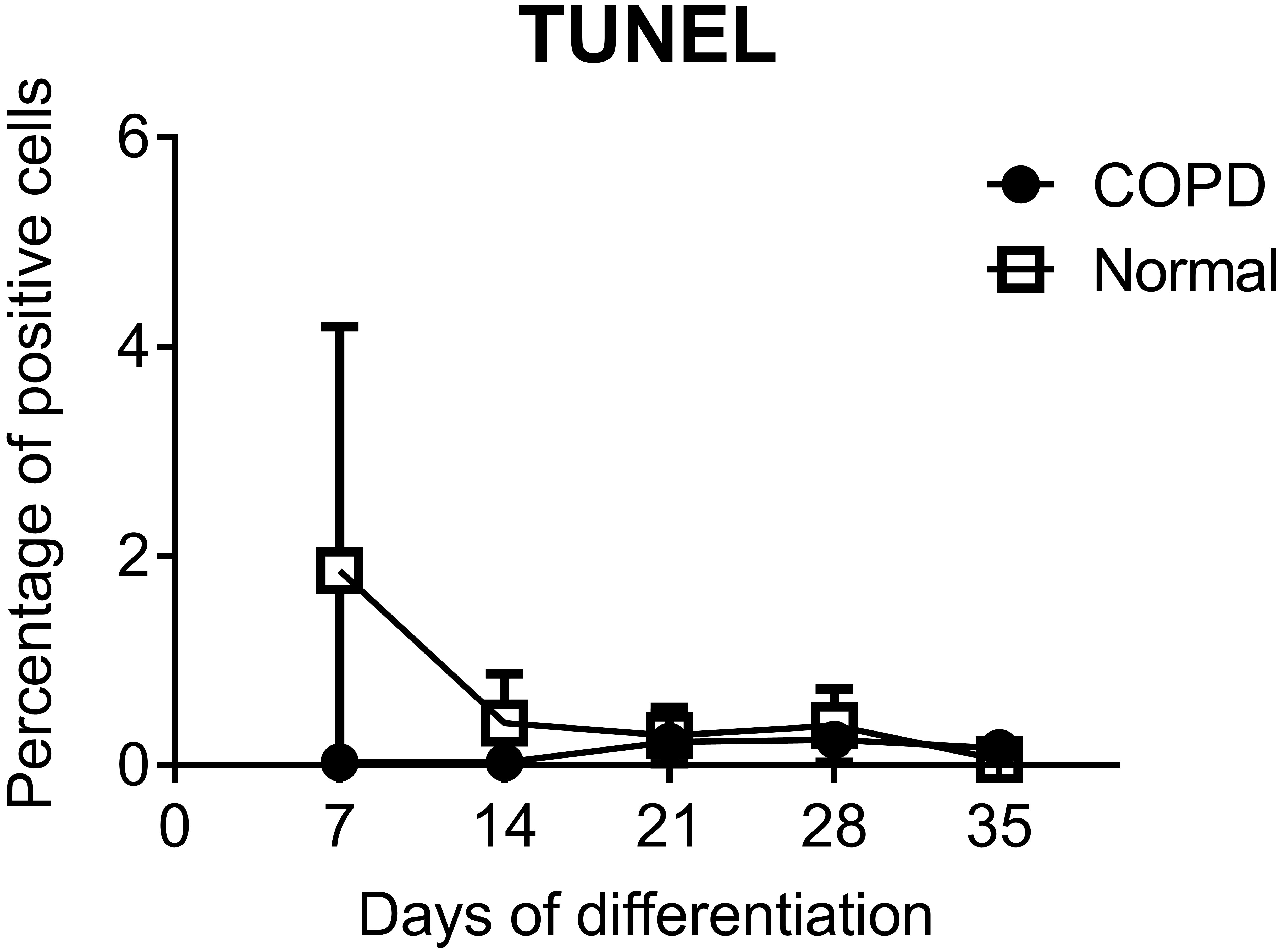


**Figure S4B. TUNEL positive primary normal human bronchial epithelial cells during differentiation on normal or COPD bronchial scaffolds** Percentages of TUNEL positive cells were calculated using image analysis of TUNEL stained tissue sections (n=3). Counterstaining with hematoxylin allowed for normalization against total number of cells. Data were analyzed with a two-way ANOVA test using Sidak correction. TUNEL=TdT-mediated dUTP Nick-End Labeling. TdT= Terminal Deoxynucleotidyl Transferase. The graph indicate mean and standard deviation.

Figure S5


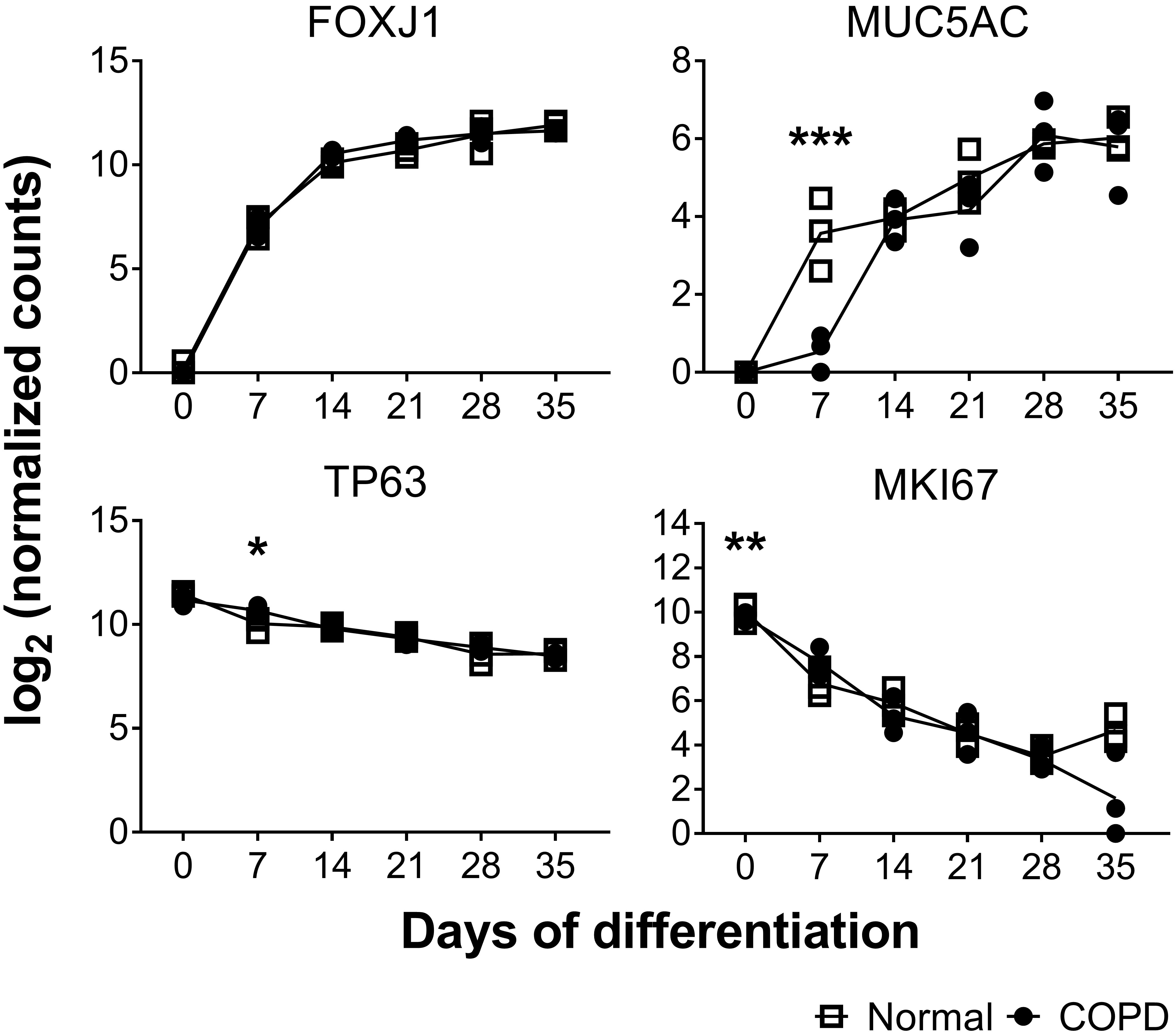


**Figure S5. Expression of FoxJ1, MUC5AC, p63 and Ki-67 mRNA in primary normal human bronchial epithelial cells on normal or COPD scaffolds.** RNA-Seq confirmed the overall expression pattern of the markers used for immunohistochemistry (IHC) (fig. 4) (n=3). However, MUC5AC mRNA was downregulated and p63 mRNA upregulated at day 7 in cells on COPD scaffolds, which was not reflected in the protein expression according to the IHC results. Ki-67 mRNA was also downregulated at day 0 on COPD scaffolds. TP63=p63, MKI67=Ki-67. The data were analyzed with DESeq2 (described in Materials and methods) using the Benjamini-Hochberg method for multiple testing correction. *FDR (False Discovery Rate)<0.05, **FDR<0.01, ***FDR<0.001.

Figure S6


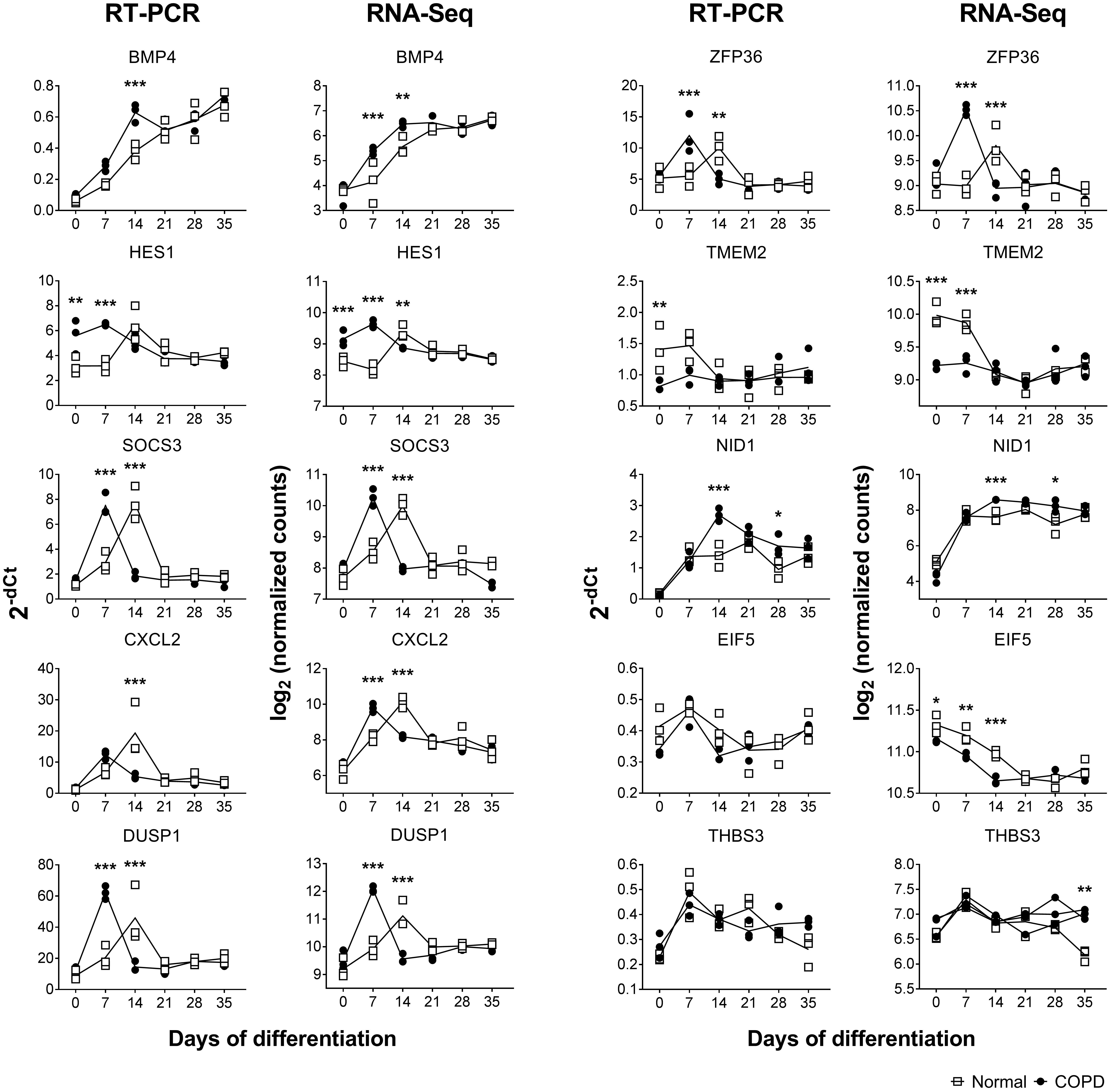


**Figure S6. Real-time quantitative RT-PCR validation of RNA-Seq data.** Real-time quantitative Reverse Transcription PCR (qRT-PCR) was used to validate the RNA-Seq data and the results aligned well between the two methods (n=3). BMP4=Bone morphogenetic protein 4, HES1=Hes family bHLH transcription factor 1, SOCS3=Suppressor of cytokine signaling 3, CXCL2=C-X-C motif chemokine ligand 2, DUSP1=Dual specificity phosphatase 1, ZFP36=ZFP36 ring finger protein, TMEM2=Transmembrane protein 2, NID1=Nidogen 1, EIF5=Eukaryotic translation initiation factor 5, THBS3=Thrombospondin 3. Real-time qRT-PCR data was normalized against expression of TBP (TATA box binding protein) mRNA and subsequently analyzed with two-way ANOVA tests using Sidak correction. RNA-Seq data were analyzed with DESeq2 (described in Materials and methods) using the Benjamini-Hochberg method for multiple testing correction. *p<0.05, **p<0.01, ***p<0.001 for real-time qRT-PCR data and *FDR (False Discovery Rate) <0.05, **FDR<0.01, ***FDR<0.001 for RNA-Seq data.

Figure S7


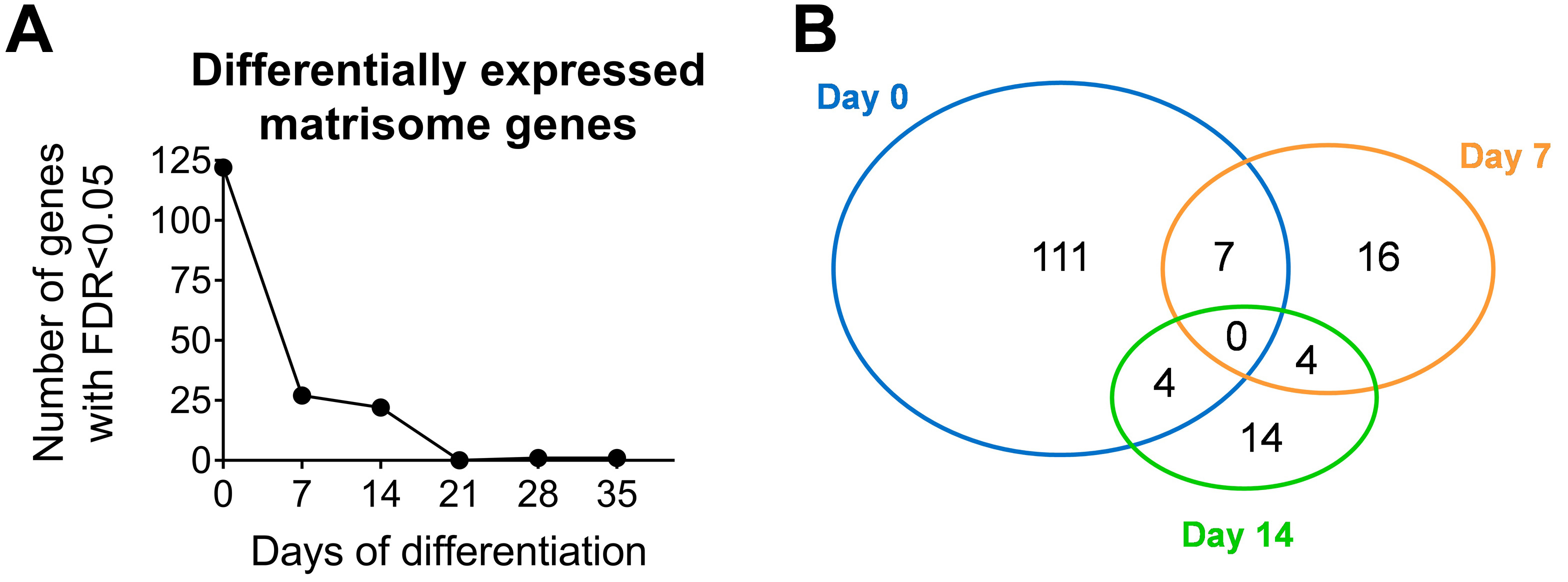


**Figure S7. Differential expression of matrisome genes in primary normal human bronchial epithelial cells differentiated on COPD compared to normal bronchial scaffolds.** **(A)** The number of differentially expressed matrisome genes in cells on COPD compared to normal bronchial scaffolds at each time point during differentiation. **(B)** The number and overlap of differentially expressed matrisome genes on day 0, 7 and 14. All data in this figure represent n=3.
